# LincRNA 1700020I14Rik alleviates cell proliferation and fibrosis in diabetic nephropathy via miR-34a-5p/Sirt1/HIF-1α signaling

**DOI:** 10.1038/s41419-018-0527-8

**Published:** 2018-04-27

**Authors:** Ailing Li, Rui Peng, Yan Sun, Handeng Liu, Huimin Peng, Zheng Zhang

**Affiliations:** 1 Molecular Medicine and Cancer Research Center, Chongqing Medical University, 400016 Chongqing, China; 2 Department of Pathology, The People’s Hospital of Rongchang Distrct, 402460 Chongqing, China; 3 Department of Bioinformatics, Chongqing Medical University, 400016 Chongqing, China

## Abstract

Long intergenic noncoding RNAs (lincRNAs) have been gradually identified to be functional in a variety of different mechanisms associating with development and epigenetic regulation of cellular homeostasis. However, the study of lincRNAs in diabetic nephropathy (DN) is still in its infancy. Here, we have found dysexpressed long noncoding RNAs (lncRNAs) in renal tissues of db/db DN mice compared with db/m mice by RNA sequencing. In this study, 5 lincRNAs were confirmed to express in a consistent trend among these DN-related lncRNAs both in vivo and in vitro. Particularly, 1700020I14Rik was the downregulated one. Moreover, our data showed overexpression or knockdown of 1700020I14Rik could regulate cell proliferation and fibrosis in mouse mesangial cells (MCs). Furthermore, 1700020I14Rik was found to interact with miR-34a-5p via both the directly targeting way by bioinformatic investigation and luciferase assay and the Ago2-dependent manner by RIP assay. Results also displayed that overexpression of 1700020I14Rik inhibited cell proliferation and expressions of renal fibrosis markers through miR-34a-5p/Sirt1/HIF-1α pathway in MCs under high glucose condition, while knockdown of 1700020I14Rik could increase cell proliferation and expressions of renal fibrosis markers. In conclusion, these results provide new insights into the regulation between 1700020I14Rik and miR-34a-5p/Sirt1/HIF-1α signaling pathway during the progression of DN.

## Introduction

Diabetic nephropathy (DN) as one of the major microvascular complications of diabetes is the primary cause of end-stage renal disease. It is typically characterized by renal proliferation, thickening of basement membranes, interstitial fibrosis, and massive accumulation of the extracellular matrix^1^. However, the detailed mechanism of DN is still unknown to date. Increasing evidence shows that silent information regulator T1 (Sirt1) is involved in many important biological processes, including inflammation, renal interstitial fibrosis, autophagy under hypoxia condition, ageing, and oxidative stress^2^. In recent years, researchers also have focused on the relationship between Sirt1 and DN. A drop of Sirt1 expression was found in DN and knockdown of Sirt1 expression could abolish the beneficial effect of an active component against renal damage in DN^3–6^, while activating Sirt1 reversed the inflammation and fibrosis induced by high glucose in rat glomerular mesangial cells (MCs) and silencing Sirt1 could promote fibrosis factors and inflammation factors in high glucose-cultured rat glomerular measangial cells through promoting its downstream molecule hypoxia-inducible factor-1α (HIF-1α) expression^7^. However, the potential regulation mechanism in Sirt1/HIF-1α signaling in DN remains to be fully elucidated.

Noncoding RNAs, arbitrarily separated into long noncoding RNAs (lncRNAs) (>200 nucleotides) and small ncRNAs (≤200), are involved in multifarious physiological and pathological progress. In the past decades, small ncRNAs, such as microRNAs (miRNAs), have been intensely investigated and found to regulate various disease progresses, some of which are further treated as therapeutic targets. However, the functional role of lncRNAs remains little known. The lncRNAs, a large class of pervasive transcriptions in the genome with poor or no protein-coding ability, have been found to participate in multifarious physiological and pathological progress^8,9^. Long intergenic noncoding RNAs (lincRNAs) have been gradually identified to be functional in a variety of different mechanisms associating with development and epigenetic regulation of cellular homeostasis. Although, its molecular mechanisms are largely unknown, numerous lncRNAs are reported to be involved in various kinds of diseases and oncogenesis, including DN^10–13^.

By using RNA sequencing (RNA-seq), we found a number of lncRNAs differently expressing in mouse model of DN, in which one of the dysexpressed lincRNAs-1700020I14Rik (ENSMUST00000147425) stood out and was chosen for further research. Previous researches have demonstrated that many lncRNAs function as competing endogenous RNA (ceRNA) and competitively bind miRNAs to affect its function and prevented its targeted transcript from being degraded^14–16^. Therefore, we propose that 1700020I14Rik would function as a ceRNA to regulate the miRNAs in DN.

In the present study, the expression level of 1700020I14Rik was decreased in DN in vivo and in vitro as detected by real-time quantitative PCR (qRT-PCR). In addition, overexpression of 1700020I14Rik inhibited cell proliferation and fibrosis in MCs, while 1700020I14Rik knockdown induced the proliferation and fibrosis of MCs with low glucose. Moreover, 1700020I14Rik was confirmed to interact with miR-34a-5p by bioinformatic analysis, dual-luciferase reporter assay, and RIP. Furthermore, data showed that 1700020I14Rik increased Sirt1 expression via competitively binding miR-34a-5p. Additionally, 1700020I14Rik affected on cell proliferation and fibrosis of MCs by Sirt1/HIF-1α signaling pathway. Taken together, our results indicate that 1700020I14Rik may play an important regulatory role in DN through miR-34a-5p/Sirt1/HIF-1α signaling.

## Results

### LincRNAs were dysexpressed in DN in vivo and in vitro, including 1700020I14Rik

**Fig. 1:**
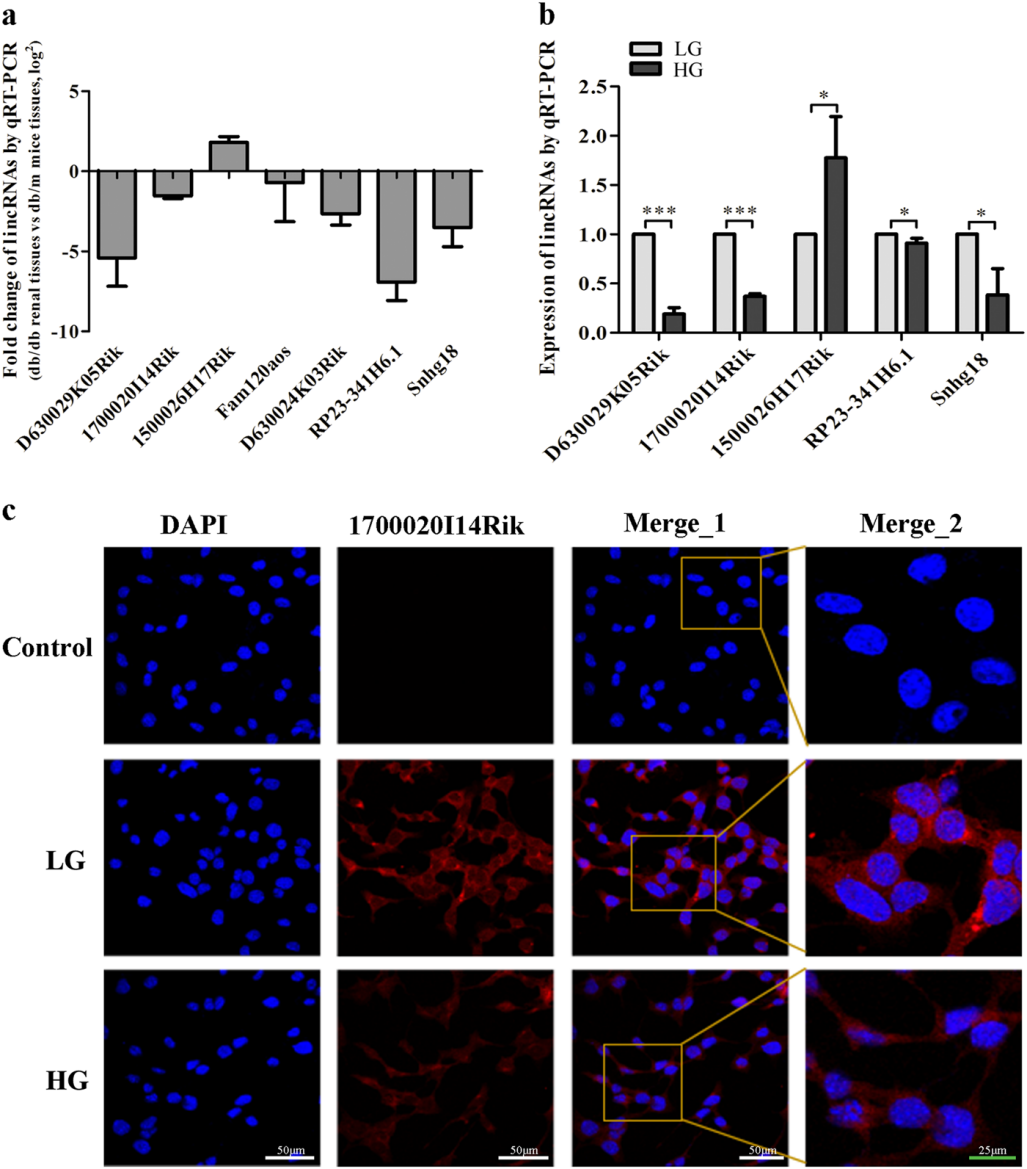
Differentially expressed lincRNAs in renal tissues or MCs and characterization of 1700020I14Rik in MCs. **a** Seven lncRNAs were validated in renal tissues by qRT-PCR and shown in consistent expression with RNA-Seq results. **b** Seven lncRNAs were further validated in MCs cultured with high or low glucose by qRT-PCR and 5 lincRNAs were in consistent with the previous results. **c** Expression of 1700020I14Rik was showed with confocal FISH images and its mainly cytoplasmic localization in MCs and highly expressed in MCs culture with high glucose medium. White scale bar, 50 µm. Green scale bar, 25 µm. Data were represented as mean ± SD. Significance was calculated using the Student’s *t*-test: *p < 0.05, **p < 0.01, and ***p < 0.001

Our study showed 106 lncRNAs were abnormally expressed in renal tissues of db/db DN mice by RNA-seq using the following criteria: *p*-value < 0.05 and *q*-value < 0.05 (Supplementary Table 1). On the basis of these data, we found there were 42 lincRNAs among these DN-related lncRNAs. Furthermore, the expressions of 7 lincRNAs with both high abundance in two groups (FPKM > 5) and fold changes >0.5 among them were verified in renal tissue of mice by qRT-PCR. Data showed that five lincRNAs showed an expression of consistent with the result of RNA-seq, including four downexpressed and one upexpressed lincRNAs (Fig. 1a). Moreover, these five lincRNAs were verified in MCs by qRT-PCR and data showed consistent results with those in renal tissues (Fig. 1b).

Then, lincRNA 1700020I24Rik (ENSMUSG00000085438; Refseq NR_027832) was focused on for further study because it was not only significantly downexpressed in DN in vivo and in vitro, but also displayed the highest conservative factor on the sequence comparing with their homologous sequence in human and high abundance (FPKM > 11) among these five lincRNAs (Supplementary Table 2). Furthermore, FISH results confirmed that 1700020I24Rik was decreased in high glucose-cultured MCs compared with that in MCs cultured with low glucose medium. Data also showed that since 1700020I24Rik located in both cytoplasm and nuclear in MCs, it was mainly distributed in the cytoplasm of cells (Fig. 1c). Therefore, these demonstrate that 1700020I24Rik may be a DN-related factor.

### Overexpression of 1700020I14Rik inhibits proliferation and fibrosis in MCs under high glucose condition

**Fig. 2:**
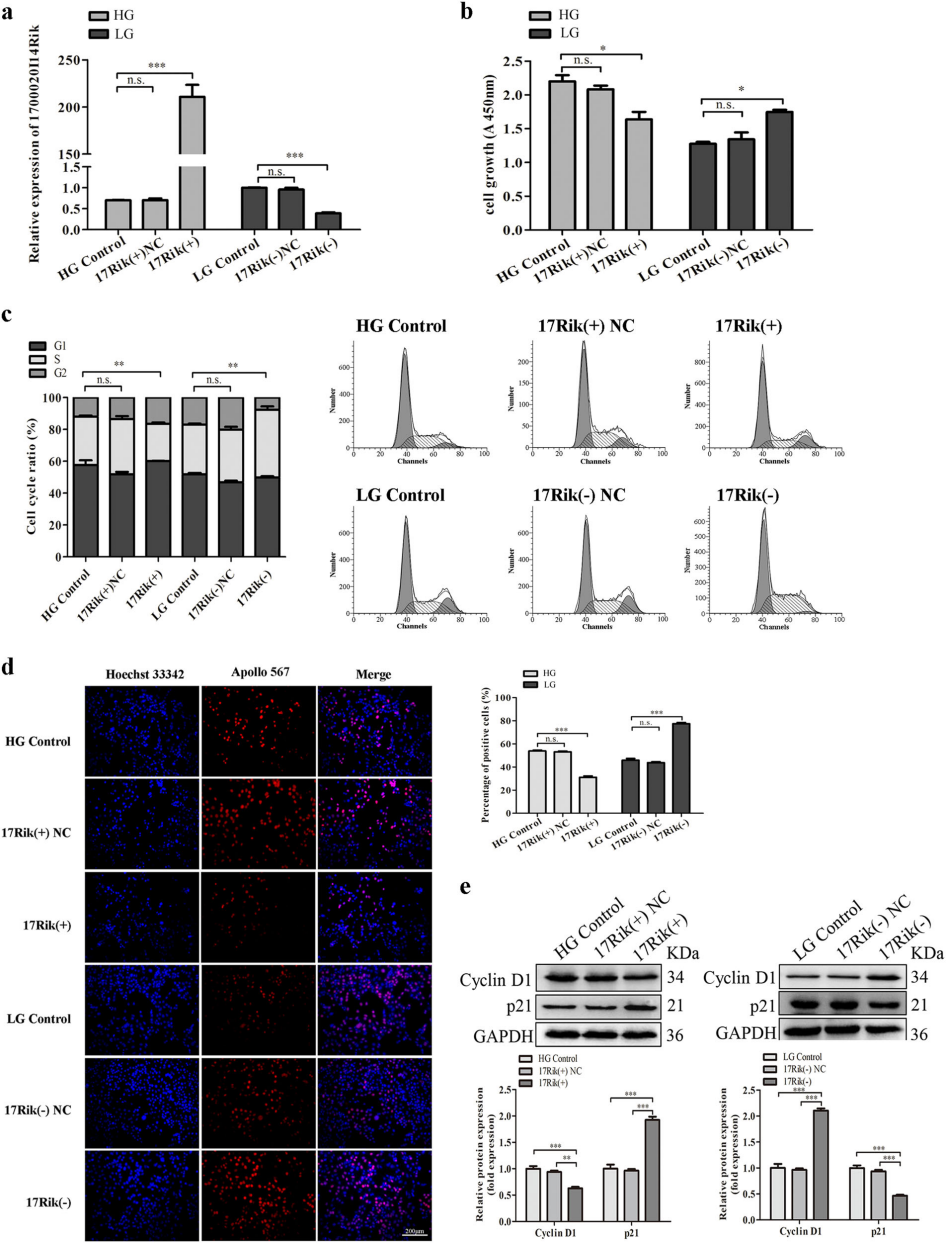
1700020I14Rik suppressed the proliferation of MCs. **a** The expression of 1700020I14Rik was measured by qRT-PCR in cells transfected with 1700020I14Rik (+) or siRNA-832 or their matched controls, which showed obviously upregulating or downregulating the expression of 1700020I14Rik compared with their matched controls, respectively. **b** CCK8 assay was performed to evaluate the effect of 1700020I14Rik on the proliferation in MCs after 48 h transfection, which suggested 1700020I14Rik could suppressed the proliferation of MCs cultured with high glucose medium. **c** Flow cytometric assay was performed to analyze the effect of 1700020I14Rik on the cell cycle in MCs transfected with 1700020I14Rik (+), siRNA-832, and their matched controls, respectively. Results showed that 1700020I14Rik regulated the S phase. **d** Proliferating mesangial cells were labeled with EdU. Scale bar, 200 µm. **e** The expressions of cell-cycle-related proteins cyclin D1 and p21 regulated by 1700020I14Rik were analyzed by western blot. Data were represented as the mean ± SD of three independent experiments. *p < 0.05, **p < 0.01, and ***p < 0.001, *n.s*: no statistical significance

To study the effects of 1700020I14Rik in DN, 1700020I14Rik overexpression or knockdown was performed to investigate its functions. Firstly, the interfering efficiency of three siRNAs against 1700020I14Rik was measured by qRT-PCR. Data suggested that siRNA-832 emerged the best knockdown efficiency, which was chosen for the following research. MCs transfected with siRNA-832 or its matched control were named as 17Rik (−) or 17Rik (−) NC, respectively. Results also showed that MCs transfected with pcDNA3.1 (+)-1700020I14Rik or siRNA-832 could effectively increase or decrease the expression of 1700020I14Rik (Fig. 2a). Moreover, CCK-8 assay showed the proliferation of MCs was decreased in 17Rik (+) group compared with that in 17Rik (+) NC or HG control groups, while the cell proliferation was enhanced in 17Rik (−) group compared with that in 17Rik (−) NC or LG control groups (Fig. 2b). Furthermore, cell-cycle analysis by flow cytometry showed that cells in the 17Rik (+) group were arrested in the S phase when compared with cells in the 17Rik (+) NC and HG control groups, whereas the proportion of cells in S phase was observably increased in 17Rik (−) compared with 17Rik (−) NC or LG control groups (Fig. 2c). In addition, EdU assay showed that cell proliferation was significantly inhibited in the 17Rik (+) group of MCs under high glucose condition compared with that in17Rik (+) NC and HG control groups, while 1700020I14Rik knockdown showed the opposite effect in the cells cultured with low glucose (Fig. 2d). Consistent with these results, we observed decreased expression of the cell-cycle-related proteins cyclin D1, and increased expression of p21 in the 17Rik (+) group, while the expression of cyclin D1 and p21 was reversed respectively once the expression of 1700020I14Rik was knockdown in the 17Rik (−) group (Fig. 2e). Therefore, these data indicate that overexpression of 1700020I14Rik inhibits cell proliferation in DN.

**Fig. 3:**
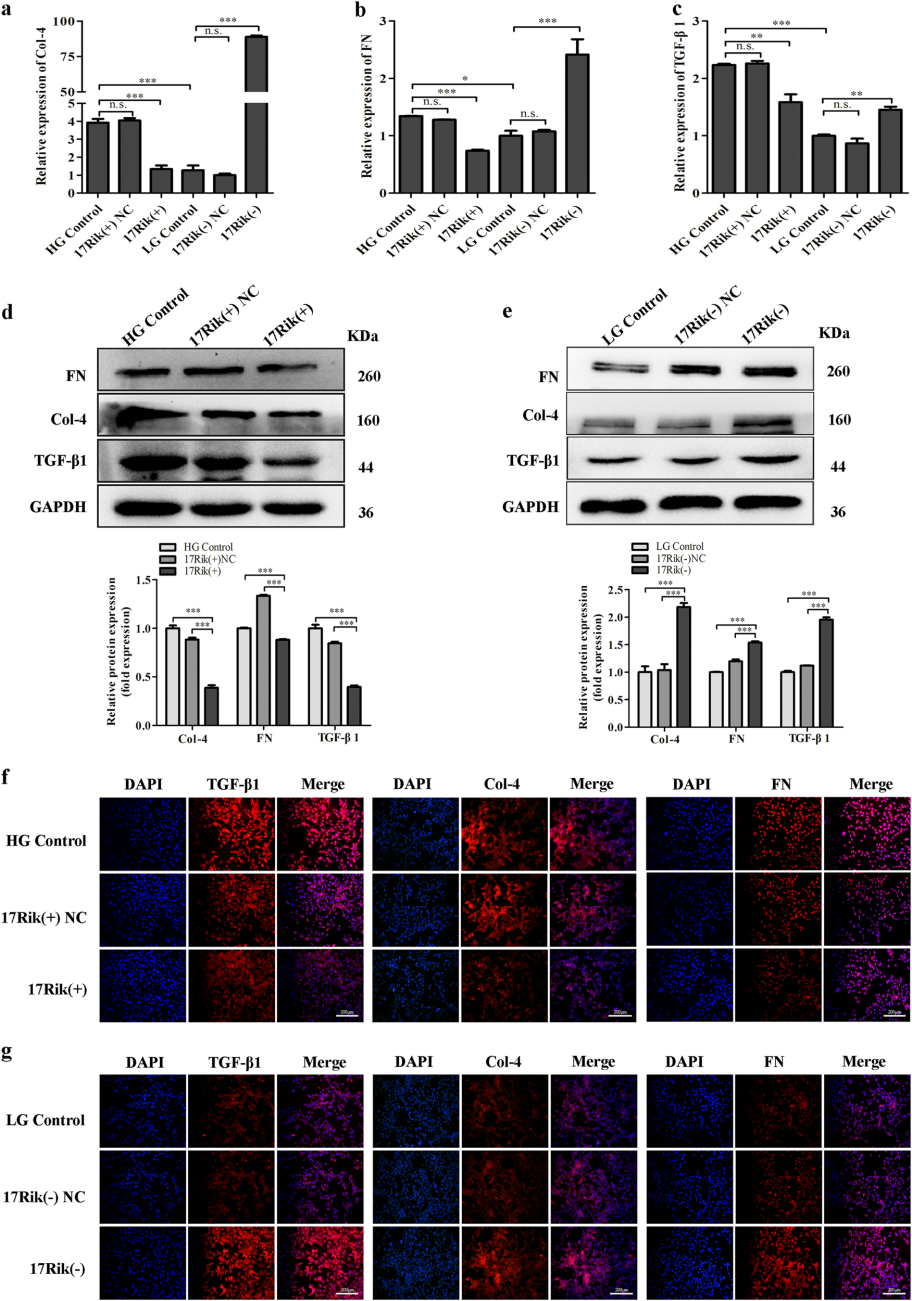
Effects of 1700020I14Rik on the regulation of fibrosis-related proteins. **a**–**c** The mRNA expression level of Col-4 (**a**), FN (**b**), and TGF-β1 (**c**) in MCs transfected with plasmids or siRNA of 1700020I14Rik was measured by qRT-PCR. **d**–**e** The protein expression level of Col-4, FN, and TGF-β1 in MCs transfected with plasmids (**d**) or in MCs transfected with siRNA-832 (**e**) was analyzed by western blot. **f**, **g** Abnormal expressions of Col-4, FN, and TGF-β1 in MCs transfected with plasmids (**f**) or in MCs transfected with siRNA (**g**) were assessed by immunofluorescence analysis. Scale bar, 200 µm. Overall, these results suggested that 1700020I14Rik alleviated renal fibrosis in DN. Data were represented as the mean ± SD of three independent experiments. *p < 0.05, **p < 0.01, and ***p < 0.001, *n.s*: no statistical significance

To explore the effect of 1700020I14Rik on fibrosis in MCs, the expression levels of the well-known renal fibrosis-related factors, collagen IV (Col-4), fibronectin (FN), and transcriptional regulatory factor-beta 1 (TGF-β1), were detected in this study. QRT-PCR data displayed that overexpression of 1700020I14Rik significantly decreased the mRNA expressions of Col-4, FN, and TGF-β1, while 1700020I14Rik knockdown increased the expressions of Col-4, FN, and TGF-β1 (Fig. 3a–c). Meanwhile, western blot analysis showed the expressions of Col-4, FN, and TGF-β1 decreased in 17Rik (+) group compared with that in the 17Rik (+) NC and HG control groups (Fig. 3d). However, the expressions of Col-4, FN, and TGF-β1 enhanced in 17Rik (−) group compared with that in 17Rik (−) NC or LG control groups (Fig. 3e). Furthermore, immunofluorescence assay revealed that expressions of Col-4, FN, and TGF-β1 reduced in 17Rik (+) group compared with that in the 17Rik (+) NC and HG control groups (Fig. 3f). However, the expressions of Col-4, FN, and TGF-β1 augmented in 17Rik (−) group compared with that in 17Rik (−) NC or LG control groups (Fig. 3g). Besides, ELISA assay also identified that overexpression of 1700020I14Rik significantly inhibited the production and activation of TGF-β1, whereas, 1700020I14Rik knockdown increased its production and activation (Supplementary Fig 1). Overall, these data suggest that 1700020I14Rik alleviates renal fibrosis in DN.

### 1700020I14Rik interacts with miR-34a-5p by both directly targeting and Ago2-dependent manners

**Fig. 4:**
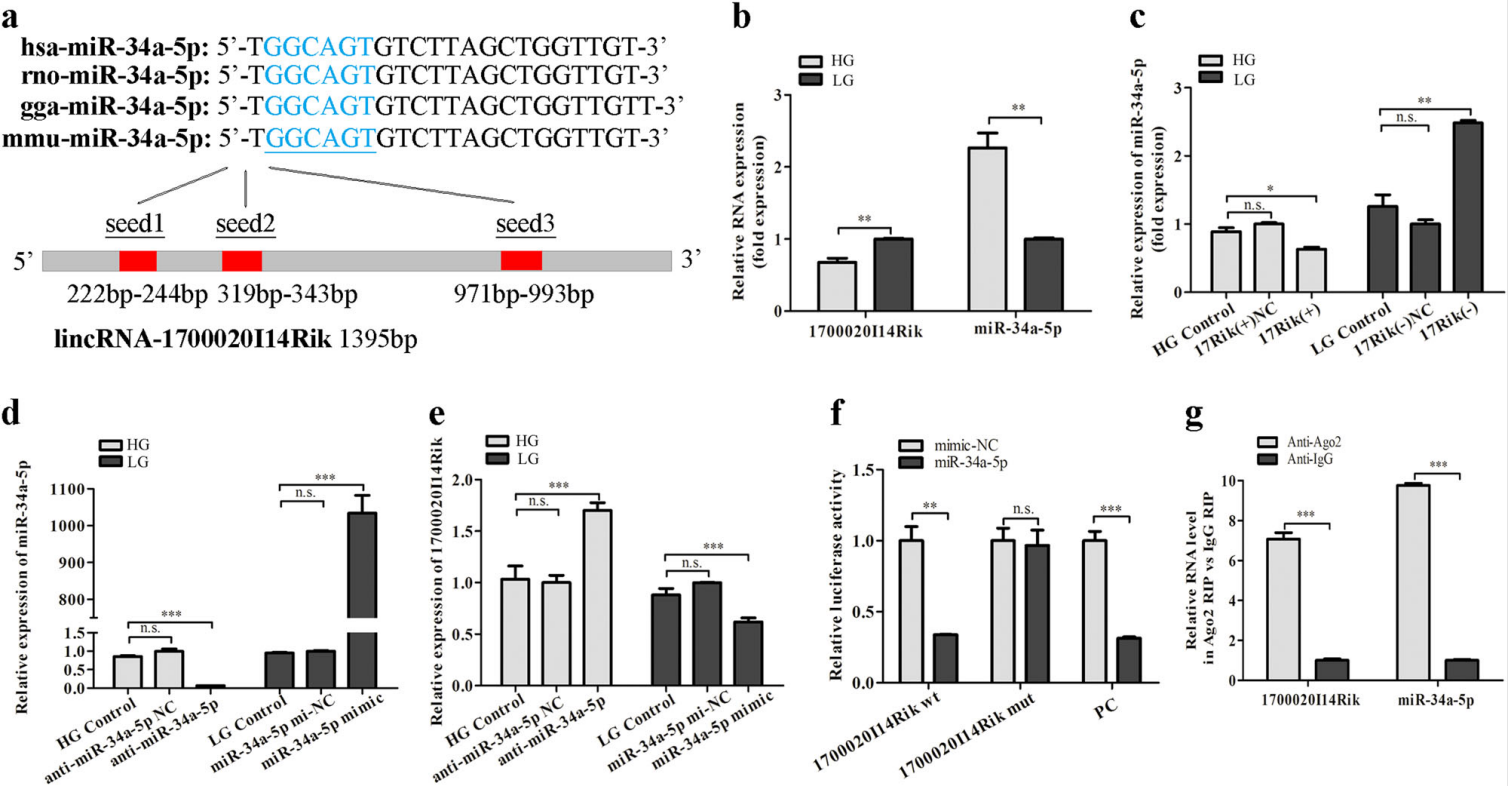
1700020I14Rik interacted with miR-34a-5p by both directly targeting and Ago2-dependent manner. **a** Predicted binding sites of miR-34a-5p of four species on 1700020I14Rik transcript. The red part of the column indicates the potential binding sites. **b** The inverse expression of 1700020I14Rik and miR-34a-5p in MCs cultured in high glucose or low glucose medium was assessed by qRT-PCR. **c** The expression of miR-34a-5p regulated by 1700020I14Rik plasmid or its siRNA was measured by qRT-PCR. **d** The expression of miR-34a-5p regulated by miR-34a-5p mimics and inhibitor was measured by qRT-PCR. **e** The expression of 1700020I14Rik regulated by miR-34a-5p mimics or inhibitor was detected by qRT-PCR. Data suggested that 1700020I14Rik had an opposite expression comparing with miR-34a-5p in MCs. **f** Dual-luciferase reporter assay was performed to measure the luciferase activity in cells co-transfected with miR-34a-5p and luciferase reporters containing 1700020I14Rik (1700020I14Rik wt), mutant transcript (1700020I14Rik mut), or miR-34a-5p inhibitor (as positive control, PC). These results indicated that there was direct interaction between 1700020I14Rik and miR-34a-5p. Data were presented as the relative ratio of firefly luciferase activity to renilla luciferase activity. **g** RIP assay was performed in MCs cultured in high glucose, followed by qRT-PCR to detect 1700020I14Rik and miR-34a-5p associated with AGO2. Data were represented as the mean ± SD of three independent experiments. *p < 0.05, **p < 0.01, and ***p < 0.001, *n.s*: no statistical significance

To explore the mechanism of 1700020I14Rik in proliferation and renal fibrosis of DN, the functions between lncRNAs and miRNAs are focused on because ceRNA mechanism plays an important role in diseases. Ten candidates of putative targets for 1700020I14Rik were predicted by online databases (USCS, miRbase, and BiBiserv2 software) (Supplementary Table 3). Among these, miR-34a-5p was chosen for predicted candidate because not only it was reported to be a DN-related miRNA^17,18^, but also it contained three binding sites in 1700020I14Rik transcript (Fig. 4a). Moreover, qRT-PCR data showed the expression level of miR-34a-5p increased significantly in MCs cultured with high glucose, which displayed the opposite expression compared with 1700020I14Rik expression in DN (Fig. 4b). Furthermore, data showed that overexpression of 1700020I14Rik reduced the expression of miR-34a-5p compared with that in the other two HG groups, whereas 1700020I14Rik knockdown elevated miR-34a-5p expression in cells under low glucose condition (Fig. 4c). In addition, miR-34a-5p mimics or inhibitor was used to up or down the expression of miR-34a-5p in MCs cultured with high or low glucose (Fig. 4d). Data also showed the overexpression of miR-34a-5p decreased the expression of 1700020I14Rik in cells with low glucose, while downexpression of miR-34a-5p increased the expression of 1700020I14Rik in cells with high glucose (Fig. 4e). Therefore, the reciprocal regulation between 1700020I14Rik and miR-34a may play a role in DN.

To determine whether 1700020I14Rik was directly repressed by miR-34a-5p, we used a luciferase reporter plasmid containing the 1700020I14Rik cDNA wild-type (1700020I14Rik wt) or seed-sequence mutant (1700020I14Rik mut), then we performed dual-luciferase assays in HEK 293 cells. A GP-miRGLO-miR-34a-5p-inhibitor plasmid was also used in HEK 293 cells as positive control (PC). As expected, the luciferase activity of 1700020I14Rik-wt was significantly decreased by miR-34a-5p mimics, while miR-34a-5p had no influence on 1700020I14Rik-Mut. And there was an apparent reduced fluorescence activity caused in PC group by miR-34a-5p mimics (Fig. 4f). These results indicate that there is a direct interaction between 1700020I14Rik and miR-34a-5p. Additionally, miRNAs have been found to bind their targets to depress and/or degrade RNA through RNA-induced silencing complex (RISC) in an Ago2-dependent manner^19–21^. Thus, RIP assay was performed using anti-Ago2 in MCs cultured with high glucose to perform the relationship between 1700020I14Rik and Ago2. Results showed that both 1700020I14Rik and miR-34a-5p immunoprecipitated with Ago2 antibody were enhanced relative to IgG control (Fig. 4g). Taken together, these data confirm that 1700020I14Rik interacts with miR-34a-5p by both directly targeting way and Ago2-dependent manner.

### MiR-34a-5p promotes proliferation and fibrosis in MCs via Sirt1/ HIF-1α signal pathway

**Fig. 5:**
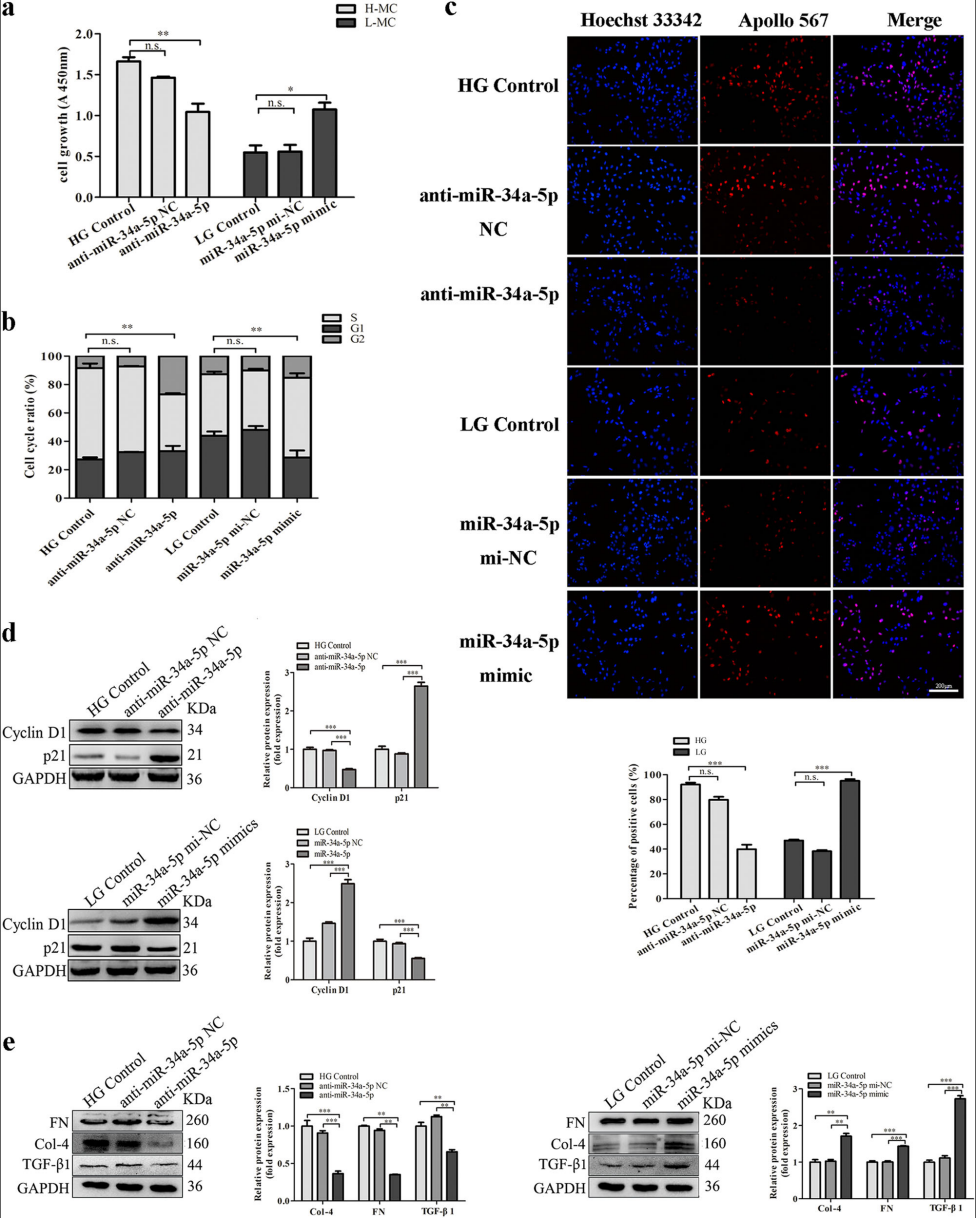
Effects of miR-34a-5p on the proliferation of MCs. **a**–**c** The proliferation of MCs transfected with miR-34a-5p mimics or inhibitor was measured by EdU assay (**a**) (Scale bar, 200 µm), by flow cytometric analysis (**b**) and by CCK-8 assay (**c**). **d**–**e** Western blot was used to analyze the expressions of cell-cycle-related proteins cyclin D1 and p21 (**d**), and the expressions of Col-4, FN and TGF-β1 regulated by miR-34a-5p (**e**). These results indicated that miR-34a-5p promotes proliferation and fibrosis in MCs. Data were represented as the mean ± SD of three independent experiments. *p < 0.05, **p < 0.01, and ***p < 0.001, *n.s*: no statistical significance

To investigate the functions of miR-34a-5p in DN, we surveyed a panel of cell proliferation and fibrosis of MCs transfected with miR-34a-5p mimics and inhibitor. The results of CCK-8 and EdU assays showed overexpression of miR-34a-5p increased cell proliferation, while inhibiting the expression of miR-34a-5p reduced its proliferation (Fig. 5a, c). Moreover, the cell-cycle analysis results showed silencing of miR-34a-5p led to an improvement of cell proportion in S phase, while overexpression of miR-34a-5p reversed the results (Fig. 5b). Furthermore, decrease-expression of miR-34a-5p reduced the expression of cyclin D1 and increased the protein level of p21, however, the expression of cyclin D1 was increased and the expression of p21 was decreased when miR-34a-5p was overexpressed (Fig. 5d). Meanwhile, overexpression of miR-34a-5p enhanced the expressions of Col-4, FN, and TGF-β1, however, inhibition of the miR-34a-5p downregulated their expressions (Fig. 5e). Therefore, the results indicate that miR-34a-5p promotes proliferation and fibrosis of MCs by regulating cyclin D1 and p21.

**Fig. 6:**
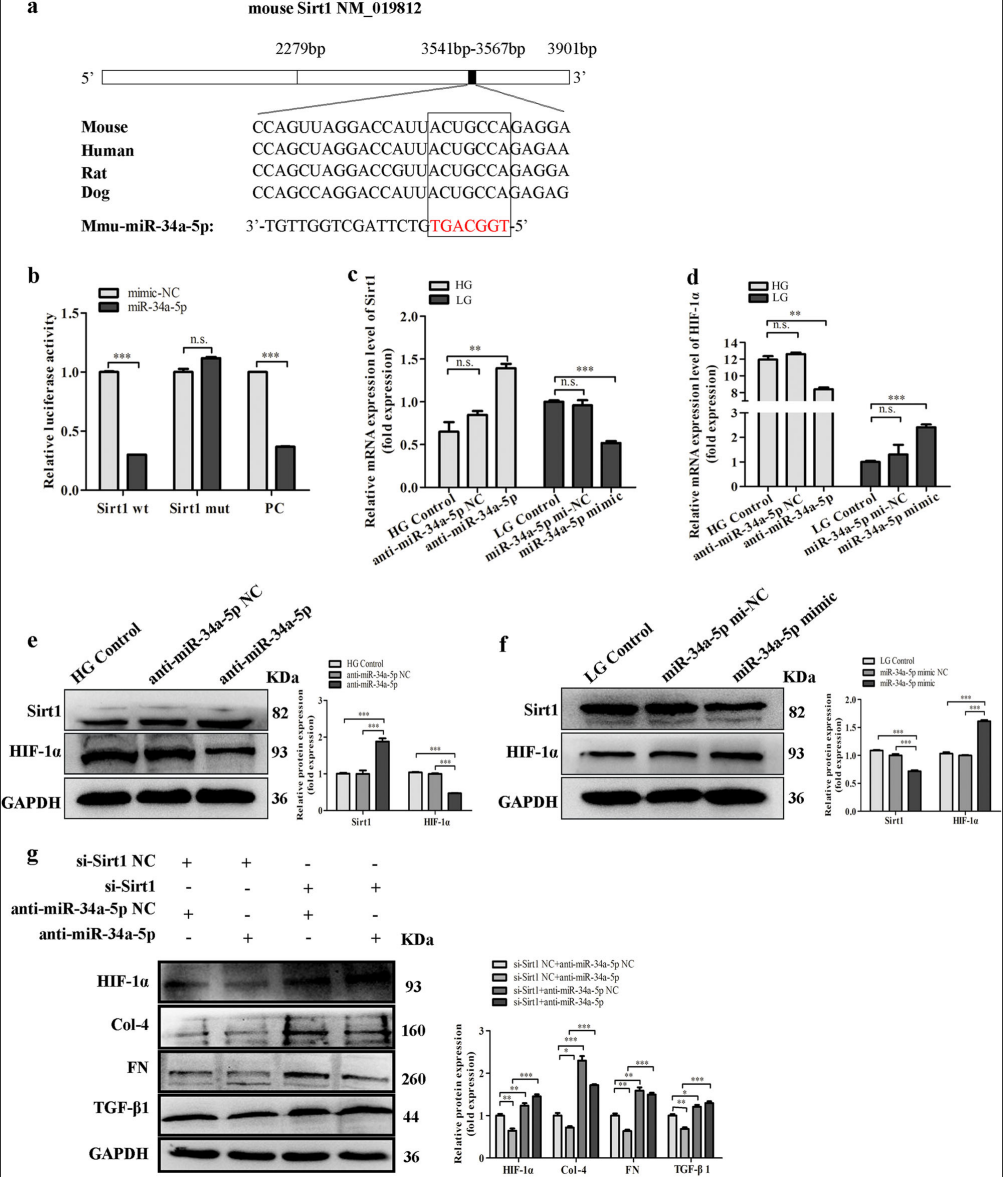
MiR-34a-5p affects the fibrosis of MCs through Sirt1/HIF-1α signaling. **a** Predicted miR-34a-5p target sequence of the Sirt1 3′-UTR showed the highly conserved binding sites in different species. The red nucleotides are the seed sequences of miR-34a-5p. **b** Dual-luciferase reporter assay was performed to measure the luciferase activity in HEK 293 cells co-transfected with miR-34a-5p and luciferase reporters containing Sirt1 (Sirt1 wt), mutant transcript (Sirt1 mut), or miR-34a-5p inhibitor (as positive control, PC). These results indicated that there was direct interaction between Sirt1 and miR-34a-5p. Data were presented as the relative ratio of firefly luciferase activity to renilla luciferase activity. **c**, **d** The mRNA expression level of Sirt1 (**c**) and HIF-1α (**d**) regulated by miR-34a-5p mimics or inhibitor was analyzed by qRT-PCR. **e–f** Protein expression levels of Sirt1 and HIF-1α regulated by miR-34a-5p inhibitor (**e**) or miR-34a-5p mimics (**f**) were measured by western blot. **g** Protein expression levels of HIF-1α, as well as its downstream proteins expression level regulated by combined effects of siRNA-Sirt1 and miR-34a-5p inhibitor were measured by western blot. These results suggested that miR-34a-5p promoted fibrosis in DN might through directly targeting to Sirt1 and regulating the expression of Sirt1. Data were represented as the mean ± SD of three independent experiments. *p < 0.05; **p < 0.01, and ***p < 0.001, *n.s*: no statistical significance

Furthermore, a highly conserved sequence was displayed that was complementary to the ‘seed sequence’ of miR-34a-5p and was identified within the Sirt1 3′-UTR by bioinformatics analysis (Targetscan) (Fig. 6a). As known, Sirt1/HIF-1α signal pathway plays an important role in the proliferation and fibrosis of DN^7^. Importantly, researchers found Sirt1 was a direct target for miR-34a-5p by luciferase assay^12,22^. In our study, we also found that miR-34a-5p could directly target to Sirt1 by luciferase assay (Fig. 6b). Moreover, our qRT-PCR and western blot analysis results showed that overexpression of miR-34a-5p significantly downregulated the expression of Sirt1 but upregulated HIF-1α in MCs with low glucose. However, the expressions of Sirt1 and HIF-1α were reversed when miR-34a-5p was silenced in MCs with high glucose (Fig. 6c–f). In addition, to further explore the relationship between miR-34a-5p and Sirt1, siRNA against Sirt1 was used and co-transfected with miR-34a-5p inhibitor in cells cultured with low glucose. Results showed the downregulation effect of miR-34a-5p inhibitor on HIF-1α was abolished by the intervention of si-Sirt1. Furthermore, western blotting showed that the effects of miR-34a-5p silence on increasing the expressions of Col-4, FN, and TGF-β1 were impeded by si-Sirt1 (Fig. 6g). Thus, the data suggest that miR-34a-5p promotes fibrosis in DN might through directly regulating the expression of Sirt1.

### 1700020I14Rik reduces the expressions of fibrosis factors in MCs by regulating miR-34a-5p/Sirt1/HIF-1α signaling

**Fig. 7:**
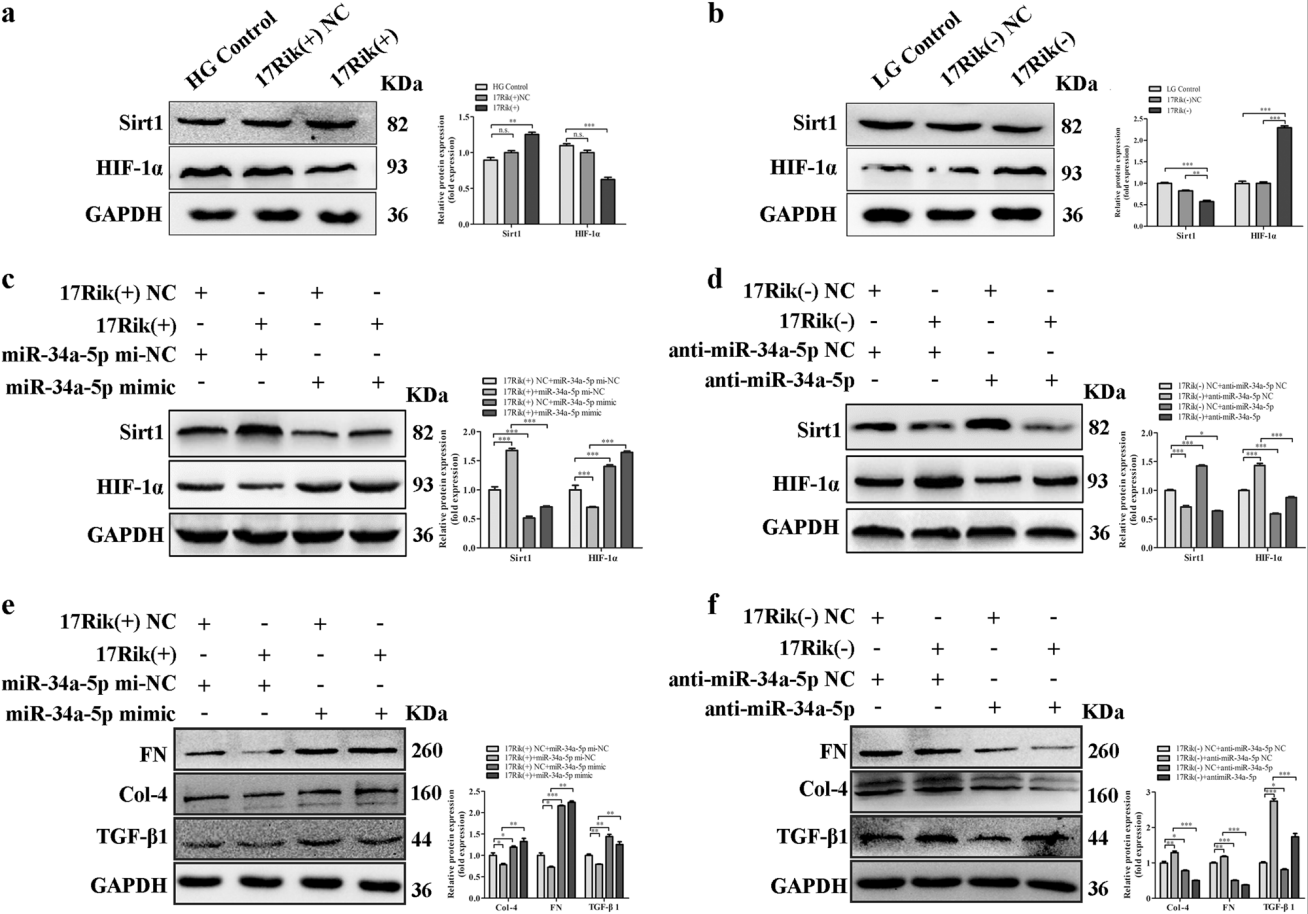
1700020I14Rik reduced the expressions of fibrosis factors in MCs by regulating miR-34a-5p/Sirt1/HIF-1α signaling. **a**–**b** Protein expression levels of Sirt1 and HIF-1α in MCs influenced by 1700020I14Rik plasmids (**a**) and by siRNA (**b**) were tested by western blot. **c**,** d** Protein expressions of Sirt1 and HIF-1α regulated by the effect of 1700020I14Rik combined with miR-34a-5p mimics (**c**) or inhibitor (**d**) were tested by western blot. These results indicated that 1700020I14Rik regulates the expressions of Sirt1 and HIF-1α by regulating miR-34a-5p. **e**, **f** Protein expression levels of Col-4, FN, and TGF-β1 regulated by the effect of 1700020I14Rik combined with miR-34a-5p mimics (**e**) or inhibitor (**f**) were measured by western blot. Results showed that the expressions of renal fibrosis genes TGF-β1, FN, and Col-4 were reduced by overexpression of 1700020I14Rik, the expressions of these proteins were abolished by the participation of miR-34a-5p mimics. The expressions of TGF-β1, FN, and Col-4 were upregulated by silence of 1700020I14Rik, these changes were blocked by miR-34a-5p inhibitor. Data were represented as the mean ± SD of three independent experiments. *p < 0.05; **p < 0.01, and ***p < 0.001, *n.s*: no statistical significance

To clarify whether the effect of 1700020I14Rik on renal fibrosis was the interaction with miR-34a-5p through Sirt1/ HIF-1α pathway, we firstly identified the effect of 1700020I14Rik on the expressions of Sirt1 and HIF-1α. Results showed that overexpression of 1700020I14Rik increased the expression of Sirt1 and reduced HIF-1α, while knockdown of 1700020I14Rik decreased the expression of Sirt1 and upregulated HIF-1α (Fig. 7a, b). Furthermore, western blotting showed that overexpression of 1700020I14Rik increased the expression of Sirt1 and reduced the expression of HIF-1α. However, these altered expressions of Sirt1 and HIF-1α were blocked by overexpression of miR-34a-5p. Meanwhile, decreased Sirt1 and upregulated HIF-1α by knockdown of 1700020I14Rik was reversed in the presence of miR-34a-5p inhibitor (Fig. 7c, d). Therefore, these results indicate that 1700020I14Rik regulates the expressions of Sirt1 and HIF-1α by regulating miR-34a-5p. Additionally, results showed that though the expressions of renal fibrosis genes TGF-β1, FN, and Col-4 were reduced by overexpression of 1700020I14Rik, the expressions of these proteins were abolished by the participation of miR-34a-5p mimics. Meanwhile, although the expressions of TGF-β1, FN, and Col-4 were upregulated by silence of 1700020I14Rik, these changes were blocked by miR-34a-5p inhibitor (Fig. 7e, f). Additionally, as known, the fibrosis-related genes collagen prolyl 4-hydroxylases 1 (P4HA1), collagen prolyl 4-hydroxylases 2 (P4HA2), and procollagen-lysine (PLOD2) are HIF-1α direct targets genes^23^. Thus, the effect of 1700020I14Rik on the expressions of P4ha1, P4ha2, and Plod2 was examined by real-time PCR in this study. Data showed that overexpression of 1700020I14Rik could decrease the expression of P4ha1, P4ha2, and Plod2. However, knockdown of the expression of 1700020I14rik increased the expression of P4ha1, P4ha2, and Plod2 (Supplementary Fig 3). Furthermore, data showed that the expression of 1700020I14Rik was decreased and the expression of miR-34a-5p was increased in the cells under high glucose, while the expressions of 1700020I14Rik and miR-34a-5p were reversed when Sirt1 was silenced (Supplementary Fig 4).

## Discussion

In the present study, we observed that a novel lincRNA 1700020I14Rik suppressed the proliferation and fibrosis of MCs cultured with high glucose medium through directly interacting with miR-34a-5p and antagonizes its function by regulating the expression of its target Sirt1 and downstream proteins. LncRNA, initially thought to be a spurious transcriptional noise^24^, was hotly studied in recent years and found to participate in the gene expression, mammalian development, and various kinds of diseases process^8,25^. According to their function roles in molecular mechanism, lncRNAs were divided into four subtypes: scaffolds, decors, guides, and signals^9^. Besides, lncRNAs could also be classified into five categories according to the location with nearby protein-coding transcripts:^26^ sense and antisense lncRNAs—overlapping one or more transcripts on the same or opposite strand, respectively; bidirectional lncRNAs—oriented head-to-head with a coding transcript within 1000 bp; intronic lncRNAs—overlapping the intron of one or more coding transcripts; and lincRNAs—do not overlap coding transcripts. In this study, we paid attention on lincRNAs, as it is easier to interpret its gene expression patterns, sequence conservation, and perturbation outcomes than those of transcripts from loci overlapping other gene classes^27^. On the basis of our RNA-seq data, 42 lincRNAs were dysexpressed in renal tissues of DN mice. Moreover, seven lincRNAs were chosen for the candidates because their high abundance and significant different expression. Our results of qRT-PCR showed five lincRNAs were in the consistent expression trend in vivo and in vitro. Interestingly, a highly abundant mammalian lincRNA, 1700020I14Rik was focused on because of the high conservative factor on the sequence comparing with their homologous sequence in human. As a homologous gene of 1700020I14Rik, OIP5-AS1was reported to reduce cell proliferation by serving as a sponge or a ceRNA for HuR in human cervical carcinoma HeLa cells^28^. Therefore, we speculated that 1700020I14Rik might also participate in cell proliferation and 1700020I14Rik might be an important factor in DN.

1700020I14Rik is a lincRNA, which locates in chromosome 2 (Chr2: 119594296–119600744). However, there is no report about 1700020I14Rik in diseases up to now. Previous studies have identified lncRNAs function mainly depending on their subcellular location. lncRNAs locating in cytoplasmic play a role in gene regulation by combining with specific proteins, and lncRNAs in nucleus mainly participated in the transcription process and chromatin remodeling^29,30^. In this study, we found that 1700020I14Rik was a mainly cytoplasmic lincRNA in MCs and it was decreased in high glucose-cultured MCs compared with that in MCs cultured with low glucose medium. High glucose condition is a key external stimulus that is extremely important for DN. Additionally, to investigate the biological function of 1700020I14Rik in DN, we assessed the cell proliferation and the expressions of renal fibrosis factors in MCs under high or low glucose condition. Our studies revealed that silencing of 1700020I14Rik promoted proliferation and fibrosis in MCs under low glucose condition, whereas overexpression of 1700020I14Rik expression led to inhibit proliferation and fibrosis in MCs under high glucose condition. Combine with the results of downexpression of 1700020I14Rik in DN, it reveals that the downexpression of 1700020I14Rik represents a potential risk for poor prognosis and contributes to DN development and progression.

Accumulating evidence has demonstrated that lncRNAs could participate in multifarious diseases progress by acting as ceRNAs to compete with miRNAs and regulate their functional role with their targets in a RISC manner. Lin et al. has demonstrated that lncRNA H19 could competitively bind miR-17-5p to regulate YES1 expression and inhibit miR-17-5p-induced cell-cycle progression, while promote migration and invasion in thyroid cancer^14^. LncRNA HOTAIR, playing an oncogenic role in gastric reported by Liu et al^15^, acted as a ceRNA that modulated the expression of human epithelial growth factor receptor 2 (HER 2) through working as a sink of miR-331-3p. Gas5 exerts tumor-suppressive functions in human glioma cells by directly targeting miR-222 and regulating the expression of its targets BMF and Plexin C1^16^. To explore the mechanism of 1700020I14Rik in DN, we focused on its target miRNAs. By online databases (USCS, miRbase and BiBiserv2 software) prediction, ten candidates of putative targets for 1700020I14Rik were predicted and miR-34-5p was chosen for further study for being a DN-related miRNA^17,18^ and containing three binding sites in 1700020I14Rik transcript. QRT-PCR data showed the expression level of miR-34a-5p displayed the opposite expression compared with 1700020I14Rik expression and there was a reciprocal regulation between 1700020I14Rik and miR-34a in DN. Dual-luciferase reporter assay was further used to explore the direct relationship between 1700020I14Rik and miR34a-5p. We identified that lincRNA 1700020I14Rik could directly bind to miR-34a-5p. Additionally, miRNAs have been found to bind their targets to depress and/or degrade RNA through RISC in an Ago2-dependent manner. In the present study, by using RIP assay, we found that lincRNA 1700020I14Rik was in a same RISC complex with miR-34a-5p. Collectively, the results display 1700020I14Rik interacts with miR-34-5p by both directly targeting and Ago2-dependent ways in DN.

Furthermore, previous microarray results have identified the overexpression of miR-34a in DN^17^. Moreover, downregulation of miR-34a could alleviate mesangial proliferation in vitro and glomerular hypertrophy in early DN mice by targeting GAS1^18^. In this study, we demonstrated that overexpression of miR-34a-5p improved the proliferation and fibrosis in mouse MCs, which played an inverse function compared with 1700020I14Rik. Moreover, recent studies showed that miR-34a could inhibit SIRT1 expression by directly binding to the 3′ UTRs of its mRNAs and effectively decreased the protein expression and inhibited the activity of SIRT1^21,31^. Sirt1, a common member of the mammalian sirtuin family, functions as a NAD^+^-dependent protein deacetylase that has protective effects against metabolic stress and plays a role in the progress of DN^32,33^. It was determined that Sirt1 inhibited FN and TGF-β1 expression in rat glomerular MCs (GMCs)^21^. Sirt1 deletion caused renal injury in diabetes through increasing the expressions of endothelin-1 and TGF-β1^31^. Reduction of Sirt1 by advanced glycation-end products (AGEs) increased FN and TGF-β1, two fibrotic indicators in GMCs, and ultimately promoted the pathological progression of DN^34^. Moreover, HIF-1α, an oxygen-sensitive monitor and regulatory protein^35^, is a downstream molecule of Sirt1 in hypoxia^36^. Previous study have revealed that silencing Sirt1 could promoted HIF-1α expression, but inhibited its downstream fibrosis factors and inflammation factors in high glucose-cultured rat glomerular measangial cells under normal oxygen condition^7^. With these views, we confirmed that miR-34a-5p could directly target to Sirt1 by luciferase assay. Furthermore, we conducted overexpression or knockdown of miR-34a, as we supposed, miR-34a-5p regulated the expression of Sirt1, HIF-1α and the downstream proteins. Moreover, there was no effects of miR-34a-5p on MCs anymore when Sirt1 was knockdown, which suggested that miR-34a-5p might promotes proliferation and fibrosis in MCs culture in high glucose through Sirt1/HIF-1α signaling pathway. On the other hand, we found that 1700020I14Rik could increase the expression level of Sirt1 but reduce HIF-1α expression, which showed an opposite regulation comparing with miR-34a-5p in MCs. Besides, the expressions of fibrosis-related HIF-1α target genes-P4ha1, P4ha2, and Plod2 were regulated by1700020I14Rik. It indicated that 1700020I14Rik was relevant with the fibrosis progress of DN. Furthermore, we performed the rescue experiment to find out the interaction between 1700020I14Rik and miR-34a-5p. These findings demonstrate that in high glucose condition, though downregulated by high glucose medium, decreased 1700020I14Rik accelerated fibrosis by increased the expression of miR-34a-5p, meanwhile, the ability of competition with the miR-34a-5p was gone down accordingly, and the expression of the miR-34a-5p target gene, Sirt1 was decreased, renal injury was further aggravated via Sirt1/HIF-1α signal mechanism in MCs.

In summary, our results indicated that high glucose medium could regulate lincRNA 1700020I14Rik and it was downexpressed in DN. Overexpression of 1700020I14Rik affected cell proliferation and fibrosis though Sirt1/HIF-1α signal pathway. Moreover, the interaction between 1700020I14Rik and miR-34a-5p via directly targeting way and Ago2-dependent manner may be the mechanism of the functions of 1700020I14Rik in DN. These data suggested a coherent mechanism for the role of 1700020I14Rik in cell proliferation and fibrosis during DN, and provided new insights into the mechanisms of renoprotection associated with1700020I14Rik and miR-34a-5p/Sirt1/HIF-1α, which could lead to a novel therapeutic strategy for DN.

## Materials and Methods

### Animals

Eight-week-old male C57BL/KsJ background db/db mice and their age-matched heterozygote male db/m mice were purchased from NBRI (Nanjing, China). Mice with genetic defects in the leptin receptor (db/db) have been widely used as type 2 diabetes model. Moreover, the db/db mouse has a long history as a model of human DN, and its key common features with the human condition are renal hypertrophy, glomerular enlargement, albuminuria, and mesangial matrix expansion^37^. Several studies established that hyperglycemia was noted at the age of 4 week and albumin excretion rates are higher by 8- to 62-fold in db/db mice beginning at the age of eight week^38^. Therefore, at eight weeks, db/db mice were regarded as early stage of DN mice^39,40^. Then, the mice were divided into two groups: CON group (db/m, *n* = 5) and DN group (db/db, *n* = 5), and significant pathological characteristics were appeared (Supplementary Fig 2). All procedures were in accordance with institutional guidelines for the care and use of laboratory animals at Chongqing Medical University. Approval of the Ethics Committee of Chongqing Medical University was obtained for the study.

### Cell culture

The mouse MCs purchased from Academy of Sciences of Shanghai (Shanghai, China) were cultured in Dulbecco’s modified eagle medium (DMEM) with 20% fetal bovine serum (FBS) at 37 °C in an atmosphere containing 5% CO_2_. According to the previous results^41^, MCs were stimulated with D-glucose at 5.5 mmol/L glucose plus 19.5 mmol/L mannitol (low glucose group, LG) or at 25 mmol/L glucose (high glucose group, HG). High glucose stimulation had imitated the growth environment of MCs under the condition of DN, and low glucose stimulation had imitated normal growth environment.

### Biochemical and histology assays

Bayer glucometer was used to measure the blood glucose and 24 h urinary albumin was measured by the coomassie brilliant blue (CBB) method. Renal tissue paraffin sections (4 µm thicknesses) subjected for hematoxylin-eosin (HE) staining were treated with hematoxylin and 1% ethanol eosin, and then mounted by neutral gum for light microscopic observation. Five random images were chosen and analyzed with Image-Pro Plus (Media Cybernetics, Bethesda, MD, USA).

### RNA extraction, library construction and sequencing

Total RNA from three db/db mice and three db/m mice were isolated using TRIzol^®^ Reagent (Life Technologies) in accordance with the manufacturer’s instructions, as described previously^42^. RNA quality and quantity were measured using an Agilent 2100 Bioanalyzer (Agilent Technologies), and then these RNA samples were used for cDNA library preparation and sequencing, which was conducted by Beijing Genomics Institute (BGI, Shenzhen, China). cDNA libraries were constructed using the TruSeq™ RNA Library Preparation Kit v2 protocol (Illumina), as described previously.^44^ cDNA libraries were sequenced using an Illumina HiSeq2000 sequencer (typical read lengths 90 bp) and 200 bp paired-end reads were generated.

### RNA-Seq reads mapping and analysis

The raw reads were firstly filtered by removing adapter sequences, low quality reads containing >50% bases with quality [QA] ≤ 15, and reads with >2% undefined nucleotides [N]. Clean reads were aligned with Ensembl Mouse Genome using Tophat (v2.0.13). Transcripts were assembled and quantified using Cufflinks (v2.2.1). FPKM (fragments per kilobase of transcript per million mapped reads) values were used to quantify the assembled transcripts. Differential expression was also tested using Cufflinks. Any transcript, with status OK and *q*-value less than 0.05, is considered as differentially expressed between the groups. The biotypes of the differentially expressed transcripts were downloaded at Ensembl.

### Plasmid transfection

The complementary sequence of 1700020I14Rik was PCR-amplified and ligated into pcDNA3.1 (+) vector (Invitrogen, US) to construct 1700020I14Rik (+) plasmid (clone loci: *BamH I*/*EcoR V*). The primers used were 5′-TCCTGGTTCTTCCATCCTGT-3′ (forward) and 5′-ACGGCTTTCCTGTGTTGAGT-3′ (reverse). Transfections were performed using opti-MEM, lipofectamine 2000, and lipofectamine 3000 reagents (Invitrogen, CA, USA) according to the manufacturer’s instructions. MiR-34a-5p mimics, inhibitors, and a matched miRNA negative control were synthesized and purified by Shanghai GenePharma Co. (Shanghai, China). Three small interfering RNAs (siRNAs) (siRNA-969, siRNA-931, siRNA-832) duplexes against 1700020I14Rik and siRNA against Sirt1 were synthesized by Shanghai Shenggong CO (Shanghai, China). MCs transfected with 1700020I14Rik (+) plasmid or pcDNA3.1 (+) plasmid were named as 17Rik (+) or 17Rik (+) NC, respectively. The transfection efficacy was measured by qRT-PCR. The sequences of plasmids used in the manuscript were shown in Supplementary Table 4.

### Reverse transcription PCR (RT-PCR) and quantitative real-time PCR (qRT-PCR)

Total RNA was extracted from MCs using RNA TRIzol (Invitrogen, US) according to the manufacturer’s instructions. The PrimeScript RT reagent Kit (Takara, Dalian, China) was used for cDNA synthesis. The quantification of miRNA, mRNA, or lncRNA was measured by qRT-PCR experiment with SYBR Premix ExTaq^TM^ II (Takara, Dalian, China) using CFX96 PCR System (Bio-Rad). Relative expressions were normalized to the expression of U6 or β-actin and calculated using 2^−ΔΔCT^ method. All experimets were performed at least three times. Primers used for PCR were listed in Supplementary Table 5.

### Fluorescence in site hybridization (FISH)

For FISH analysis, the MCs were dividied into three goups, named as the control group, which was cultured with low glucose medium and was not treated with fluorescence probe of 1700010I14Rik, the LG group, and the HG group, which were both treated with fluorescence probe but cultured with low glucose medium and high glucose medium, respectively. Each group was repeated three times. The slides were fixed in 4% paraformaldehyde (Sigma) and before pre-hybridization, cells were permeabilized with cold 0.1 % Triton X-100. Pre-hybridization buffer was discarded and hybridization was carried out with the 1700020I14Rik probe overnight. After washing with SCC buffer, the coverslip was dyed with DAPI and fluorescence test was conducted with laser scanning confocal microscope.

### Flow cytometric analysis

MCs transfected for 48 h in 6-well plates were collected, washed with PBS for three times, and resuspended in 0.5 ml PBS, fixed in 3.5 ml 70% cold ethanol at 4 °C overnight. Then, the cells were centrifuged and washed for three times with PBS, resuspended and stained in PI/Triton X-100 (20 µg PI/0.1% Triton X-100) including 0.2 mg RNase A at 37 °C. BD Flow cytometry was performed to analyze cell-cycle distribution by standard procedure.

### Cell proliferation assay

Cell proliferation was assessed using Cell Counting Kit-8 (CCK-8) (Dingguo BioTechnology Co Ltd, Beijing, China) and a 5-ethynyl-2-deoxyuridine (EdU) labeling/detection kit (RiboBio, Guangzhou, China). For CCK-8, MCs seeded in a 96-well plate at a density of 8 × 10^3^ per well were transfected with plasmids, siRNA, mimics or inhibitor for 48 h. Then, 10 µl CCK-8 regent was added to each well containing 100 µl DMEM medium and the plate was incubated for 2 h at 37 °C. A microplate reader (iMark; Bio-Rad) was used for absorbance at 450 nm.

For EdU assay, the MCs were cultured in 96-well plate and transfected with plasmids, siRNA, mimics or inhibitor for 48 h when cells at about 80% confluence. A volume of 50 µM EdU labeling medium was added into each well and cells were incubated for 2 h at 37 °C under 5% CO_2_. After fixed with 4% paraformaldehyde, the cells were treated with 0.5% Triton X-100 and washed with PBS. Subsequently, anti-EdU working solution was used to stain the cells and 100 µl of Hoechst 33342 (5 µg/ml) was added to incubate cells at room temperature. Fluorescent microscope (Olympus, Japan) was performed to observe EdU-positive cells.

### Dual-luciferase reporter assay

The 1700020I14Rik cDNA cloned into a pmiR-RB-REPORT™ dual-luciferase vector (RuiBio Corp., Guangzhou, China) to generate a pmiR-RB-REPORT™-1700020I14Rik wild-type, was named 1700020I14Rik wt. The mutant construct without predicting binding sequences of miR-34a-5p was also cloned into pmiR-RB-REPORT™ vector and named 1700020I14Rik mut. The miR-34a-5p-inhibitor oligo was cloned in GP-miRGLO dual-luciferase vector (GenePharma Co., Shanghai, China) to generate a GP-miRGLO-miR-34a-5p-inhibitor. All the recombinant plasmids were confirmed by restriction enzyme digestion and DNA sequencing. HEK 293 cells were transfected with 1700020I14Rik wt, 1700020I14Rik mut, miR-34a mimics, or mimics negative control (mimics NC) with lipofectamine 2000. HEK 293 cells were transfected with GP-miRGLO-miR-34a-5p-inhibitor, miR-34a mimics, or mimics NC with lipofectamine 2000. After 48 h, the cells were conducted with Dual-Luciferase^®^ Reporter Assay System (Promega, WI, USA). Firefly luciferase activity was normalized to Renilla luciferase activity (seen detailed procedures for Supplemental Materials).

Annealing method was conducted to generate a pair of most stable Sirt1 oligo, a pair of Sirt1 mutant oligo and miR-34a-5p-inhibitor sponge. All the sequence fragments were cloned in GP-miRGLO dual-luciferase vector (GenePharma Co., Shanghai, China), respectively, to generate a GP-miRGLO-Sirt1 wild-type (Sirt1 wt), a GP-miRGLO-Sirt1 mutant (Sirt1 mut), and a GP-miRGLO-miR-34a-5p-inhibitor. All the recombinant plasmids were confirmed by restriction enzyme digestion and DNA sequencing. HEK 293 cells were transfected with Sirt1 wt, Sirt1 mut, GP-miRGLO-miR-34a-5p-inhibitor, miR-34a mimics, or mimics NC with lipofectamine 2000. After 48 h, the cells were conducted with Dual-Luciferase^®^ Reporter Assay System (Promega, WI, USA). Firefly luciferase activity was normalized to renilla luciferase activity (seen detailed procedures for Supplemental Materials).

### ELISA Analysis

Cell lysis buffer from each treatment group was collected and detected using a mouse TGF-β1 enzyme-linked immunosorbent assay (ELISA) Kit (Boster Bio-engineering Co, Ltd, Wuhan, China), according to the manufacturer’s instruction (seen detailed procedures for Supplemental Materials).

### RNA immunoprecipitation (RIP)

To determine whether 1700020I14Rik was associated with the RNA-induced silencing complex (RISC), RIP assays were performed using the EZMagna RIP kit (Millipore, Billerica, MA, USA) and the Ago2 antibody (ab32381, Abcam, Cambridge, MA, USA) following the manufacturer’s protocol. qRT-PCR analysis was performed to measure the expression levels of 1700020I14Rik and miR-34a-5p. Nomal mouse IgG (Millipore, Billerica, MA, USA) was used as negative control (seen detailed procedures for Supplemental Materials).

### Western blot analysis

Total protein from MMCs was extracted by ice-cold RIPA lysis buffer (Beyotime, Shanghai, China). Equal amount of protein samples were subjected into 10% SDS-PAGE (Beyotime, Shanghai, China), transferred onto 0.22 µm PVDF membranes (Millipore, Temecula, CA), and blocked in 5% nonfat milk. Then the membranes were incubated with specific primary antibodies. The primary antibodies used in the study were the following: cyclin D1 (Abcam, USA, 1:10000), p21 (Boster, China, 1:400), Col-4 (Proteintech, USA, 1:1000), FN (Proteintech, USA, 1:1000), HIF-1α (Sangon,China, 1:500), Sirt1 (Sangon, China, 1:500), TGF-β1 (Proteintech, USA, 1:500), and GAPDH (Abcam, USA, 1:5000). ECL system (Millipore, Temecula, CA) was used to detect the immunoreactive bands and GAPDH antibody was used as control. Gray value of protein bands were quantified by Image Lab software.

### Immunofluorescence assay

For immunofluorescence analysis, the fixed cells were permeablized with 0.1% Triton X-100 and blocked in 5% goat serum solution, then primary antibodies FN (Proteintech, USA, 1:50), Col-4 (Proteintech, USA, 1:50), or TGF-β1 (Proteintech, USA, 1:50) was added onto the coverslips at 4 °C for overnight and Cy3-labeled secondary antibody (Proteintech, USA, 1:50) was treated onto coverslips for 1 h at room temperature. After treated with DAPI, coverslips were sealed with glycerine. The images were observed with QImaging Micro-publisher^TM^ 5.0 RTV (Olympus Corporation, Japan) and analyzed with Image-Pro Plus (Medium Cybemetics, Bethesda, MD, USA).

### Statistical analysis

Statistical analysis was analyzed by SPSS19.0. All the results were presented as means ± SD. Student’s *t*-test (two groups) and one-way ANOVA (no less than three groups) with Tukey’s Multiple Comparisons Test were used as appropriate to analyze the significance of differences between groups. A probability value *p* < 0.05 was considered statistically significant.

## Electronic supplementary material

Supplementary Information
