# Supplementary material for: LincRNA 1700020I14Rik alleviates cell proliferation and fibrosis in diabetic nephropathy via miR-34a-5p/Sirt1/HIF-1α signaling
Source: Cell Death Dis. 2018 Apr 27;9(5):461. doi: 10.1038/s41419-018-0527-8 (PMC5919933; doi:10.1038/s41419-018-0527-8)

Supplemental Materials

Dual luciferase reporter assay. The 1700020I14Rik cDNA was amplified with primers 5'-GCGCTCGAGCTTTCGCGCCTCCGCCCCT-3' (forward) and 5'-AATGCGGCCGCGGGTTGAAGGAACTTATTTATACTT-3' (reverse) and then cloned into a pmiR-RB-REPORT™ dual-luciferase vector (Guangzhou RuiBio Corp., Guangzhou, China) with restriction endonucleases *Xho I* and *Not I* to generate a pmiR-RB-REPORT™-1700020I14Rik wild-type, named 1700020I14Rik-Wt. The mutant construct without predicting binding sequences of miR-34a-5p was generated using the QuikChange™ Site-Directed Mutagenesis kit (Stratagene) and also cloned into pmiR-RB-REPORT™ vector, named 1700020I14Rik-Mut. miR-34a-5p-inhibitor oligo acquired by annealed, was cloned in GP-miRGLO dual-luciferase vector (GenePharma Co., Shanghai, China) to generate a GP-miRGLO-miR-34a-5p-inhibitor. All the recombinant plasmids were confirmed by restriction enzyme digestion and DNA sequencing. HEK 293 cells seeded in 96-well plates at a density of 4×10^3 cell per well, were transfected at a density of 80%-85% with 1700020I14Rik-Wt, 1700020I14Rik-Mut, miR-34a mimics or non-target control with lipofectamine 2000. 293T cells seeded in 12-well plates at a density of 5×10^5 cell per well, were transfected at a density of 80%-90% with GP-miRGLO-miR-34a-5p-inhibitor, miR-34a mimics or non-target control with lipofectamine 2000 for positive control used. After 48 hours, cells were conducted with Dual-Luciferase® Reporter Assay System (Promega, WI, USA). Firefly luciferase activity was normalized to Renilla luciferase activity.

Annealing method was conducted to generate a pair of most stable Sirt1 oligo (sense strand: 5'-CTAGAGCCTGCATAGATCTTCACCACAAATACTGCCAAGATGTGAATATGCAAAGCCTTTCC-3', antisense strand: 5'-TCGAGGAAAGGCTTTGCATATTCACATCTTGGCAGTATTTGTGGTGAAGATCTATGCAGGCTCTAGAGCT-3'), a pair of Sirt1 mutant oligo (sense strand: 5'-CTAGAGCCTGCATAGATCTTCTGGTCAAATTGACGGAAGATGTGAATATGCAAAGCCTTTCC-3', antisense strand: 5'-TCGAGGAAAGGCTTTGCATATTCACATCTTCCGTCAATTTGACCAGAAGATCTATGCAGGCTCTAGAGCT-3') and miR-34a-5p-inhibitor sponge (sense strand: 5'-

CACAACCAGCTAAGACACTGCCAACCGGTACAACCAGCTAAGACACTGCCAC-3’, antisense strand: 5’-TCGAGTGGCAGTGTCTTAGCTGGTTGTACCGGTTGGCAGTGTCTTAGCTGGTTGTGAGCT -3’). All the sequence fragments were cloned in GP-miRGLO dual-luciferase vector (GenePharma Co., Shanghai, China) respectively with restriction endonucleases *Xho I* and *Sca I* to generate a GP-miRGLO-Sirt1 wild-type (Sirt1-Wt), a GP-miRGLO-Sirt1 mutant (Sirt1-Mut) and a GP-miRGLO-miR-34a-5p-inhibitor (as positive control, PC). All the recombinant plasmids were confirmed by restriction enzyme digestion and DNA sequencing. 293T cells seeded in 12-well plates at a density of 5×10^5cell per well, were transfected at a density of 80%-90% with Sirt1-Wt, Sirt1-Mut, GP-miRGLO-miR-34a-5p-inhibitor, miR-34a mimics or non-target control with lipofectamine 2000. After 48 hours, cells were conducted with Dual-Luciferase® Reporter Assay System (Promega, WI, USA). Firefly luciferase activity was normalized to Renilla luciferase activity.

ELISA Analysis. The mouse MCs were seeded in 12-well plates at a density of 10^5 cell per well with DMEM medium containing 10% FBS and transfected with corresponding plasmids at a density of 80%. After 48h of treatment, cell lysis buffer Cell lysis buffer from each treatment group was collected and subjected to TGF-β1 measurement using a mouse enzyme-linked immunosorbent assay (ELISA) Kit (Boster Bio-engineering Co, Ltd, Wuhan, China), according to the manufacturer’s instruction. The absorbance was measured at 450 nm using a microplate reader (iMark; Bio-Rad). Each experiment was repeated in triplicate.

RNA immunoprecipitation (RIP). RIP assays were performed using the EZMagna RIP kit (Millipore, Billerica, MA, USA) and the Ago2 antibody (ab32381, Abcam, Cambridge, MA, USA) following the manufacturer’s protocol. Generally, MCs after treatment were collected at 95% confluency and were lysed using RIP lysis buffer. 100 μl cell lysis buffer was incubated with 50 μl RIP buffer containing magnetic beads conjugated with 5 μg mouse anti-Ago2 antibody or negative control normal mouse IgG. All samples were treated with Protein K to digest the protein and the immunoprecipitated RNA was isolated with hopper magnet. QRT-PCR was further used for analysis of 1700020I14Rik and miR-34a-5p in purified RNA.

Supplementary Fig 1. The effect of 1700020I14Rik on the production and activation of TGF-β1 determined by ELISA assay. The results found that 1700020I14Rik could evaluate TGF-β production and activation level. Data were represented as the mean ± SD of three independent experiments. Significance was calculated using Student's *t*-test: *p< 0.05; ***p< 0.001.

Supplementary Fig 2. Characteristics of qRT-PCR samples from db/db mice (n = 5) and db/m mice (n = 5). (a) Body weight, (b) Blood glucose and (c) Urinary microablumin. Statistical difference was analyzed by Wilcoxon signed-rank test (n = 10, **p < 0.01). (d) Representative morphological photographs of HE-stained kidney specimens from db/m and db/db DN mice. Scale bar, 100 μm.

Supplementary Fig 3. The effect of 1700020I14Rik on the expressions of P4ha1, P4ha2 and Plod2 was tested by real-time PCR. The results suggested that over-expression of 1700020I14Rik could decrease the expression of P4ha1, P4ha2 and Plod2. However, knockdown the expression of 1700020I14rik increased the expression of P4ha1, P4ha2 and Plod2. Data were represented as the mean ± SD of three independent experiments. **p < 0.01, ***p< 0.001.

Supplementary Fig 4. The expressions of 1700020I14Rik and miR-34-5p were tested when Sirt1 was silenced by real-time PCR. The results suggested that the expression of 1700020I14Rik was decreased and the expression of miR-34a-5p was increased in the cells under high glucose, while the expressions of 1700020I14Rik and miR-34a-5p were reversed when Sirt1 was silenced. Data were represented as the mean ± SD of three independent experiments. **p < 0.01, ***p< 0.001.

Supplementary Table 1. Differently expressed lncRNAs from RNA-seq.

gene_name	[a]gene_id	location	Average FPKM		db/db mice vs. db/m mice			biotype
			db/m mice	db/db mice	[b]log2FC	[c]p-value	q-value	
Gm20204	ENSMUSG00000098148	chr8:117210777-117215997	1.27708	0.810469	-0.656024	0.0026	0.0366798	lincRNA
Gm5101	ENSMUSG00000097814	chr1:96872220-96881230	1.5718	0.960778	-0.710147	0.001	0.0172698	antisense lncRNA
Snhg18	ENSMUSG00000096956	chr15:32240568-32244662	11.5791	6.9539	-0.735627	0.00015	0.00371321	lincRNA
4930556M19Rik	ENSMUSG00000096971	chr15:10714835-10790123	2.09827	1.20338	-0.802107	1.00E-04	0.00261518	lincRNA
AB041803	ENSMUSG00000044471	chr6:31165522-31218433	4.9634	2.63106	-0.915684	5.00E-05	0.00143789	lincRNA
9130020K20Rik	ENSMUSG00000086568	chr4:136347285-136350334	0.890854	0.462217	-0.94662	8.00E-04	0.014485	lincRNA
RP23-341H6.1	ENSMUSG00000098747	chr9:83240480-83254540	11.4813	5.4326	-1.07957	0.00085	0.0152559	lincRNA
D630029K05Rik	ENSMUSG00000031294	chr10:116956823-116972609	37.7919	17.2977	-1.12749	5.00E-05	0.00143789	lincRNA
2610037D02Rik	ENSMUSG00000097536	chr15:96134818-96284495	3.38625	1.47401	-1.19994	5.00E-05	0.00143789	lincRNA
Gm16685	ENSMUSG00000097804	chr3:7572076-7690001	3.91434	1.69891	-1.20415	5.00E-05	0.00143789	antisense lncRNA
AV099323	ENSMUSG00000087377	chr2:132253356-132261333	1.95121	0.717632	-1.44305	0.00035	0.00745285	antisense lncRNA
Gm15348	ENSMUSG00000074469	chr8:12706943-12719127	1.40459	0.486305	-1.53021	5.00E-05	0.00143789	lincRNA
Gm9951	ENSMUSG00000054618	chr8:34049261-34057046	0.760201	0.232949	-1.70637	0.00305	0.0412082	lincRNA
C330002G04Rik	ENSMUSG00000097930	chr19:23037389-23075853	1.71647	0.478632	-1.84246	5.00E-05	0.00143789	lincRNA
Snhg6	ENSMUSG00000098234	chr1:9908637-9944118	11.3401	2.53366	-2.16213	0.00015	0.00371321	lincRNA
Gm15354	ENSMUSG00000086156	chr8:65617899-66471637	1.90016	0.328395	-2.53261	0.00175	0.0267907	antisense lncRNA
2610035D17Rik	ENSMUSG00000087259	chr11:113043894-113201838	4.75221	0.808922	-2.55453	0.00015	0.00371321	lincRNA
4930533I22Rik	ENSMUSG00000093535	chr6:57476696-57497671	2.38779	0.397161	-2.58788	5.00E-05	0.00143789	antisense lncRNA
C920006O11Rik	ENSMUSG00000097574	chr9:78175913-78178879	1.90179	0.0972766	-4.28912	0.00065	0.0122617	lincRNA
Gm15611	ENSMUSG00000084923	chr5:8998401-8999669	1.27588	0	-	5.00E-05	0.00143789	lincRNA
C030037D09Rik	ENSMUSG00000087574	chr11:88718644-88728893	0.366867	1.71025	2.22088	5.00E-05	0.00143789	lincRNA
Gm26652	ENSMUSG00000097497	chrX:19232962-19237856	0.847693	3.22343	1.92698	0.00385	0.0490365	lincRNA
1600020E01Rik	ENSMUSG00000097048	chr6:86526270-86564442	1.49555	5.04878	1.75526	5.00E-05	0.00143789	lincRNA

Gm2115	ENSMUSG00000097789	chr7:84528974-84578337	0.563199	1.8337	1.70304	5.00E-05	0.00143789	lincRNA
Gm4419	ENSMUSG00000097055	chr12:21417910-21419803	0.507754	1.45123	1.51507	5.00E-05	0.00143789	lincRNA
Gm12962	ENSMUSG00000087171	chr4:131647047-131677845	1.30304	3.53361	1.43927	6.00E-04	0.0114831	lincRNA
Gm13855	ENSMUSG00000087613	chr6:34179231-34186392	9.30575	24.0819	1.37175	5.00E-05	0.00143789	antisense lncRNA
Gm15706	ENSMUSG00000086013	chr6:145250528-145251849	1.28697	3.30528	1.3608	5.00E-05	0.00143789	lincRNA
D630024D03Rik	ENSMUSG00000085772	chr11:31790112-31824524	3.3665	7.77048	1.20675	5.00E-04	0.00996939	lincRNA
AW822252	ENSMUSG00000085998	chrX:53706027-53716578	0.873162	1.89283	1.11623	0.0027	0.0377186	lincRNA
1700007L15Rik	ENSMUSG00000097318	chr16:33379854-33380727	1.609	3.40984	1.08354	0.00105	0.0179263	lincRNA
6030443J06Rik	ENSMUSG00000097207	chr5:22550436-22807850	0.440961	0.832906	0.917505	0.0012	0.019969	lincRNA
2810001G20Rik	ENSMUSG00000087497	chr11:64079483-64083259	3.88733	7.2219	0.893598	2.00E-04	0.00475763	antisense lncRNA
Gm15343	ENSMUSG00000085468	chr10:76562271-76570532	6.20418	11.4231	0.880639	5.00E-05	0.00143789	antisense lncRNA
1500026H17Rik	ENSMUSG00000097383	chr10:89686370-89700866	1.41735	2.56977	0.858443	1.00E-04	0.00261518	lincRNA
Fam169b	ENSMUSG00000074071	chr7:68266338-68363089	2.16081	3.67712	0.767003	4.00E-04	0.00830783	lincRNA
Fam120aos	ENSMUSG00000097059	chr13:48968286-48972031	11.2115	16.3793	0.546893	0.00145	0.0232134	lincRNA
1700020I14Rik	ENSMUSG00000085438	chr2:119594296-119607502	16.1698	11.2289	-0.526083	0.00145	0.0232134	lincRNA
2310009A05Rik	ENSMUSG00000098332	chr9:73039717-73042775	43.9643	24.8009	-0.82594	5.00E-05	0.000579745	antisense lncRNA
2610507I01Rik	ENSMUSG00000085882	chr11:59197792-59202431	5.35443	0.238232	-4.4903	5.00E-05	0.000579745	processed transcript
2700038G22Rik	ENSMUSG00000097180	chr5:23850596-23855033	0.267309	0.895358	1.74396	0.00435	0.0224804	lincRNA
2810001G20Rik	ENSMUSG00000087497	chr11:64079483-64083259	4.62402	8.58899	0.893341	0.00015	0.00147348	antisense lncRNA
2810002D19Rik	ENSMUSG00000045464	chr2:94406706-94411682	1.7149	2.56812	0.582589	0.02655	0.0903879	processed transcript
2810008D09Rik	76972	chr11:117076782-117078955	8.60657	12.0774	0.488803	0.0396	0.121904	lincRNA
2810408I11Rik	ENSMUSG00000087213	chr1:64679868-64690659	2.36703	3.77629	0.673893	0.0349	0.110949	antisense lncRNA
3000002C10Rik	ENSMUSG00000070282	chr9:109830152-109831431	0.995344	0.483584	-1.04143	0.0232	0.0817409	pseudogene
3110045C21Rik	ENSMUSG00000097503	chr1:169969408-170088944	8.73402	4.57137	-0.934019	0.03045	0.100552	antisense lncRNA
4921504A21Rik	ENSMUSG00000097626	chr5:19202367-19226555	1.34522	0.883659	-0.606276	0.03655	0.114753	antisense lncRNA
5530601H04Rik	ENSMUSG00000087174	chrX:105040853-105070124	1.5729	1.01884	-0.62649	0.00845	0.0378529	processed transcript

5930430L01Rik	ENSMUSG00000106951	chr5:148990055-148995215	2.07893	1.29259	-0.685575	0.00185	0.011279	lincRNA
A330035P11Rik	ENSMUSG00000085615	chr14:122097938-122106974	1.09838	0.655455	-0.744808	0.01075	0.0456911	processed transcript
A930005H10Rik	ENSMUSG00000054426	chr3:115881577-115888130	7.41662	12.0386	0.698837	0.00255	0.0147033	antisense lncRNA
AA543186	ENSMUSG00000097345	chr2:25327449-25332571	0.527142	0.908351	0.785057	0.04445	0.132933	lincRNA
AI506816	ENSMUSG00000105987	chr5:23692260-23712667	0.251955	1.92944	2.93694	0.02565	0.0882092	processed transcript
B330016D10Rik	ENSMUSG00000048406	chr4:141546161-141548313	1.92736	3.3823	0.811377	0.00035	0.00294773	pseudogene
BC039771	ENSMUSG00000087594	chr2:145701194-145753672	0.618325	0.266728	-1.21299	0.0262	0.0895789	processed transcript
C330006A16Rik	ENSMUSG00000087679	chr2:26136806-26140506	23.1832	31.5343	0.443843	0.0054	0.0266045	lincRNA
D330023K18Rik	ENSMUSG00000087269	chr2:31150247-31152291	3.12338	8.73258	1.4833	0.00025	0.00227238	processed transcript
E130307A14Rik	ENSMUSG00000087177	chr10:39612565-39732007	1.25916	0.192889	-2.70662	0.00855	0.0381935	antisense lncRNA
Gm10052	ENSMUSG00000058922	chr9:123688847-123690275	7.23171	4.348	-0.733984	0.00035	0.00294773	pseudogene
Gm12669	ENSMUSG00000100863	chr4:91805549-91806555	94.5234	128.114	0.43868	0.00555	0.0271839	pseudogene
Gm13483	ENSMUSG00000085862	chr2:50296809-50433967	2.22821	1.10878	-1.00691	0.01385	0.0556058	processed transcript
Gm15455	ENSMUSG00000081402	chr1:33835898-33838914	1.21857	1.76485	0.534352	0.0227	0.0805309	pseudogene
Gm15772	ENSMUSG00000062353	chr5:3236389-3236928	187.155	297.465	0.66849	5.00E-05	0.000579745	pseudogene
Gm1943	384864	chr8:109339786-109340908	1.21044	2.21209	0.869881	0.00965	0.04207	pseudogene
Gm1966	ENSMUSG00000073902	chr7:106595548-106644645	1.15791	0.484274	-1.25762	5.00E-05	0.000579745	pseudogene
Gm1976	ENSMUSG00000066057	chr17:94750099-94834799	0.70401	0.35611	-0.983273	0.02775	0.0936335	lincRNA
Gm3219	100041231	chr14:34345054-34345686	14.8814	28.9356	0.959339	5.00E-05	0.000579745	pseudogene
Gm3716	ENSMUSG00000105402	chr5:64593862-64610699	4.15758	5.58791	0.426564	0.0215	0.0771146	lincRNA
Gm4956	ENSMUSG00000025936	chr1:21275326-21298312	6.81471	11.1787	0.714024	0.0004	0.00328891	pseudogene
Gm5176	382421	chr10:111500787-111501345	2.13233	0.949424	-1.16731	0.0281	0.0944546	pseudogene
Gm6300	ENSMUSG00000104375	chr3:14363116-14377566	11.6027	0.0173284	-9.38711	0.0478	0.14019	pseudogene
Gm6548	ENSMUSG00000091549	chr17:78850503-78852544	13.3029	17.756	0.416562	0.01605	0.0623654	pseudogene
Lhx1os	ENSMUSG00000087211	chr11:84525659-84535831	6.97024	2.96946	-1.23101	0.00035	0.00294773	lincRNA
Gm33354	ENSMUSG00000104194	chr1:168119363-168432258	7.76237	1.84097	-2.07603	0.02015	0.0736309	TEC

Gm38197	ENSMUSG00000102460	chr3:30124494-30126616	2.20294	1.01046	-1.12442	5.00E-05	0.000579745	TEC
9230112E08Rik	ENSMUSG00000070461	chr2:180719119-180721976	9.30088	6.84957	-0.441353	0.0123	0.0507109	Sense overlapping
Gm15459	ENSMUSG00000100801	chr5:5781529-5783636	50.3944	86.4201	0.778104	0.0402	0.123442	pseudogene
Nlrp5-ps	ENSMUSG00000041596	chr7:14530653-14623066	2.13547	3.34631	0.648016	0.00665	0.0313764	pseudogene
Cyp2j15-ps	ENSMUSG00000084286	chr4:96243453-96258269	1.93227	0.515422	-1.90647	5.00E-05	0.000579745	pseudogene
Mir99ahg	ENSMUSG00000090386	chr16:77236561-77691644	3.30948	1.59069	-1.05695	0.04895	0.143011	TEC
4833413G10Rik	ENSMUSG00000104950	chr5:3571659-3637101	7.08017	3.9065	-0.857907	0.0315	0.102974	antisense lncRNA
1700056N10Rik	ENSMUSG00000084792	chr16:17047612-17049204	1.65269	0.96332	-0.778728	0.03485	0.110879	lincRNA
Gm15638	ENSMUSG00000085826	chr16:45945718-46010413	7.53584	3.87033	-0.96131	0.00185	0.011279	antisense lncRNA
Gm10145	ENSMUSG00000064208	chr10:27936241-27937484	2.33233	3.50418	0.587304	0.0228	0.0808138	pseudogene
Gm37315	ENSMUSG00000104095	chr1:20052150-20055629	3.40376	1.16444	-1.5475	5.00E-05	0.000579745	TEC
Gm6300	ENSMUSG00000104375	chr3:14363116-14377566	0.882403	0.00922159	-6.58028	0.0479	0.140406	pseudogene
Gm11548	ENSMUSG00000085320	chr9:90089965-90114820	1.27231	0.588932	-1.11128	0.0023	0.0134515	lincRNA
Gm16316	ENSMUSG00000087129	chr8:46146055-46150772	0.769038	0.437436	-0.813984	0.0224	0.0796617	antisense lncRNA
Gm16685	ENSMUSG00000097804	chr6:57426690-57447665	1.20844	0.259869	-2.21729	0.0003	0.00262508	antisense lncRNA
Gm37728	ENSMUSG00000103220	chr6:31095127-31097419	1.33324	0.554745	-1.26504	0.00035	0.00294773	TEC
Gm38197	ENSMUSG00000102460	chr8:8665074-8690537	22.9808	12.3346	-0.897717	0.00605	0.0291405	TEC
Gm15348	ENSMUSG00000074469	chr8:12706943-12719127	1.58841	0.572112	-1.47321	5.00E-05	0.000579745	lincRNA
1810019N24Rik	ENSMUSG00000097115	chr7:13533940-13547117	4.75035	1.06972	-2.15079	0.0057	0.0277736	lincRNA
4833413G10Rik	ENSMUSG00000104950	chr5:3525611-3530998	2.60754	1.37191	-0.9265	0.00105	0.0071877	antisense
1700040K01Rik	ENSMUSG00000103840	chr16:4473364-4475211	0.86773	0.451433	-0.942736	0.0175	0.0663975	TEC
Gm37509	ENSMUSG00000104379	chr1:15805657-15846580	0.922429	0.448186	-1.04134	0.01015	0.0437947	TEC
AA914427	ENSMUSG00000103149	chr16:17922135-17923301	0.639275	1.19391	0.90119	0.0331	0.106717	TEC
Gm6483	ENSMUSG00000087153	chr8:19682024-19698079	1.75602	1.23938	-0.502685	0.0412	0.125641	pseudogene
Chic2	ENSMUSG00000029229	chr5:75006423-75044626	0.936926	0.406495	-1.2047	0.02665	0.0906703	Retained intron
Gm37014	ENSMUSG00000102838	chr1:137953757-137956010	1.12446	0.71012	-0.663098	0.02785	0.0938517	TEC

Gm16316	ENSMUSG00000087129	chr2:163491686-163541721	3.44931	1.8037	-0.935346	0.00035	0.00294773	antisense lncRNA
Plekhd1os	ENSMUSG00000044062	chr12:80686374-80692466	0.228388	0.696568	1.60878	0.0358	0.112932	antisense lncRNA
Ppp6r1	ENSMUSG00000052296	chr7:4631494-4658950	26.3844	33.2771	0.334842	0.03535	0.111889	pseudogene
Slc2a4rg-ps	ENSMUSG00000085028	chr2:181384249-181387596	1.72336	2.64362	0.617287	0.00555	0.0271839	pseudogene
Zfp862-ps	ENSMUSG00000107476	chr6:48504338-48534832	0.642447	0.436432	-0.557821	0.02705	0.0916985	pseudogene

[a]Ensembl gene IDs.

[b]log2FC:log2 fold change.

[c]q-value cut-off is 0.05.

[d]p-value cut-off is 0.05.

[e]biotype from Ensembl.

Supplementary Table 2. Characteristics of candidate lincRNAs.

lincRNA name	Conservative factor	Average FPKM		Regulation
		db/db mice	db/m mice	
D630029K05Rik	N/A	37.7919	17.2977	down
1700020I14Rik	54%	16.1698	11.2289	down
1500026H17Rik	51%	1.41735	2.56977	up
RP23-341H6.1	N/A	11.4813	5.4326	down
Snhg18	16%	11.5791	6.9539	down

Supplementary Table 3. The alternative miRNAs predicted to target on 1700020I14Rik transcript.

microRNA	NO. of targeting sites	ΔG
Mmu-miR-124-5p	2 (1025bp-1047bp; 1192bp-1214bp)	-26.4
Mmu-miR-135a-5p	1 (355bp-378bp; 367bp-391bp)	-21.7
Mmu-miR-195a-5p	1 (881bp-903bp)	-19.4
Mmu-miR-195b	1 (881bp-903bp)	-20.4
Mmu-miR-215-3p	1 (77bp-90bp; 1063bp-1084bp)	-31.1
Mmu-miR-221-3p	1 (917bp-940bp)	-32.5
Mmu-miR-34a-3p	1 (41bp-62bp)	-24.6
Mmu-miR-34a-5p	3 (222bp-244bp; 319bp-343bp; 971bp-993bp)	-20.5
Mmu-miR-377-3p	1 (112bp-137bp)	-26.4
Mmu-miR-690	1 (1190bp-1211bp)	-25.3

Supplementary Table 4. The alternative plasmids sequences used in the manuscript.

Plasmid name	Strand	Sequence (5’-3’)
miR-34a-5p mimic	Sense	UGGCAGUGUCUUAGCUGGUUGU
	Anti-sense	AACCAGCUAAGACACUGCCAUU
SiRNA-969	Sense	GGACAAUGCUCACCCUGAATT
	Anti-sense	UUCAGGGUGAGCAUUGUCCTT
SiRNA-961	Sense	GCCUGUGGCUAGUUCAGAATT
	Anti-sense	UUCUGAACUAGCCACAGGCTT
SiRNA-832	Sense	GGAGAAGAAACCUGUUGUUTT
	Anti-sense	AACAACAGGUUUCUUCUCCTT
SiRNA-Sirt1	Sense	GGGAUCAAGAGGUUGUUAATT
	Anti-sense	UUAACAACCUCUUGAUCCCTT
miR-34a-5p inhibitor		ACAACCAGCUAAGACACUGCCA

Supplementary Table 5. Primers used for qPCR.

Gene ID	Primer	Sequence (5’-3’)
1700020I14Rik	Forward	TCCTGGTTCTTCCATCCTGT
	Reverse	ACGGCTTTCCTGTGTTGAGT
Sirt1	Forward	TGTCTCCTGTGGGATTCCTGACTTC
	Reverse	TGGCTTGAGGGTCTGGGAGGT
HIF-1α	Forward	GGAGATCCTTCGAGGAGCACTT
	Reverse	GGCGATTTAGCAGCAGATATAAGAA
Col-4	Forward	GAACTGGCAGAATCGGGACAG
	Reverse	TCCAATGGGACCCTTATCTCC
FN	Forward	ATGTGGACCCCTCCTGATAGT
	Reverse	GCCCAGTGATTTCAGCAAAGG
TGF-β1	Forward	TGACGTCACTGGAGTTGTACGG
	Reverse	GGTTCATGTCATGGATGGTGC
β-actin	Forward	ATATCGCTGCGCTGGTCGTC
	Reverse	AGGATGGCGTGAGGGAGAGC

U6	Forward	CTCGCTTCGGCAGCACA
	Reverse	AACGCTTCACGAATTTGCGT
P4ha1	Forward	AAGGCTGAGCCGAGCTACA
	Reverse	GCCAAGCACTCTTAGATACTCTG
P4ha2	Forward	TTGCTAAGCCCAAACTTGCAC
	Reverse	GCATCTTCGTCATCGCTCCT
Plod2	Forward	GAGAGGCGGTGATGGAATGAA
	Reverse	ACTCGGTAAACAAGATGACCAGA
Mmu-miR-34a-5p	RT	CTCAACTGGTGTCGTGGAGTCGGCAATTCAGTTGAGACAACCAG
	Forward	ACACTCCAGCTGGGTGGCAGTGTCTTAGC
	Reverse	CTCAACTGGTGTCGTGGA

Supplementary Fig 1.

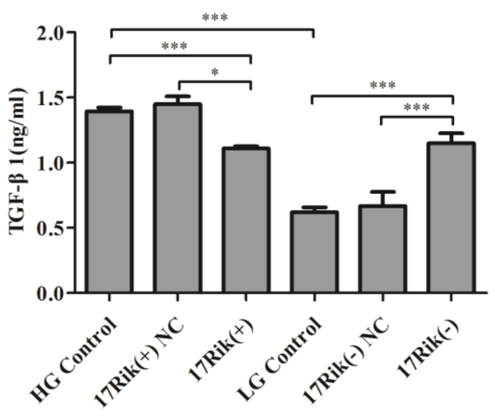

Supplementary Fig 2.

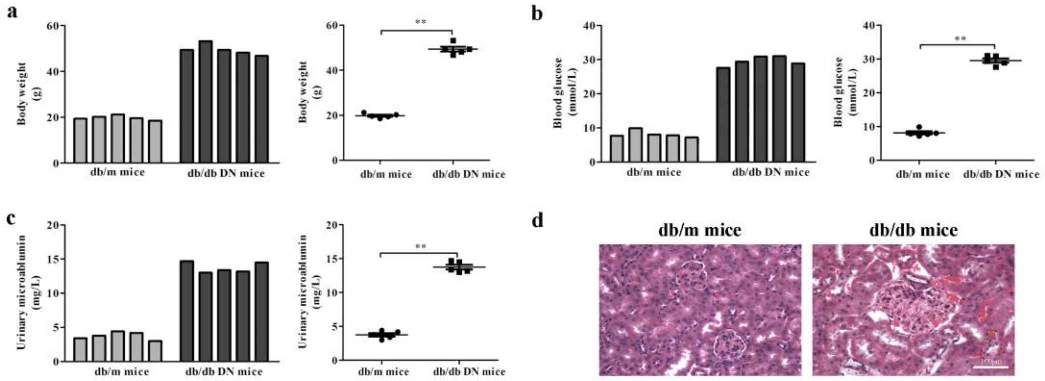

Supplementary Fig 3.

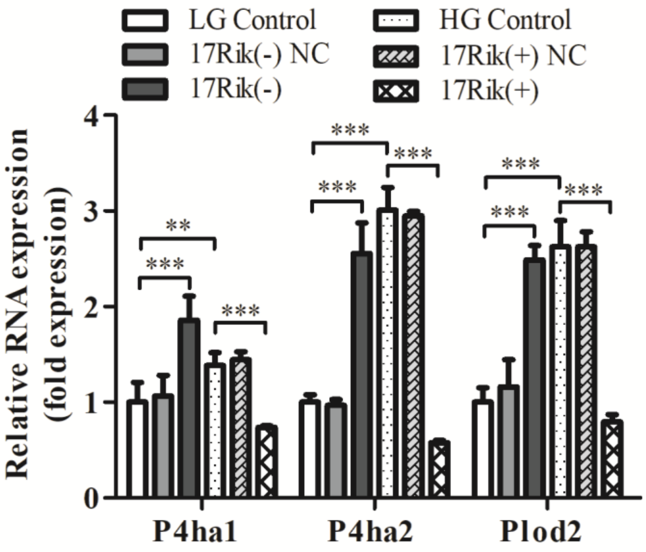

Supplementary Fig 4.

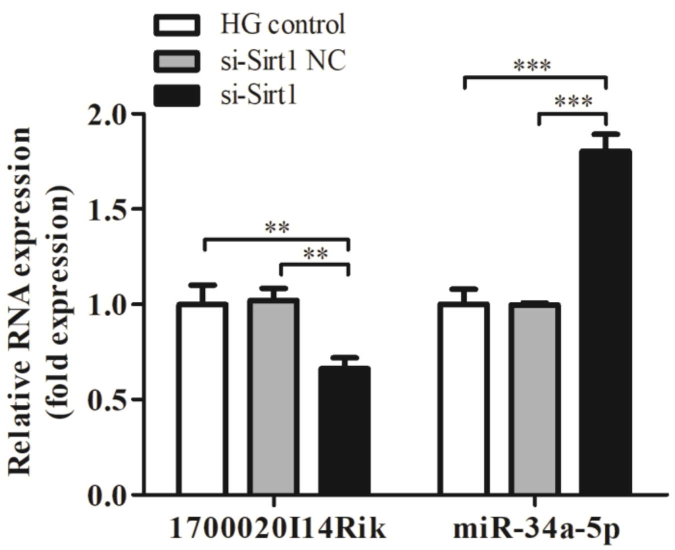

Supplement: Supplementary file 1 — Supplementary Information [file 41419_2018_527_MOESM1_ESM.pdf]
